# Effects of Macroprudential Policies on Bank Lending and Credit Risks

**DOI:** 10.1007/s10693-022-00378-z

**Published:** 2022-03-24

**Authors:** Stefanie Behncke

**Affiliations:** Swiss National Bank, Berne, Switzerland

**Keywords:** Banks, Countercyclical capital buffer, Financial stability, Loan-to-value ratio, Macroprudential policy, Mortgages, E5, G21, G28

## Abstract

I analyse the effects of two macroprudential policy measures implemented in Switzerland: the activation of the countercyclical capital buffer (CCyB) and a cap on the loan-to-value (LTV) ratios. I use a difference-in-differences method to estimate the effects of these measures on risk indicators, such as their LTV and loan-to-income (LTI) ratios and mortgage growth rates. I find that both the CCyB and the LTV cap led to a reduction in high LTV mortgages. The banks affected by the CCyB also reduced their mortgage growth rates. I do not find any evidence that these measures had unintended consequences on LTI risks, other measures of mortgage lending standards, or non-mortgage credit growth.

**Supplementary Information:**

The online version contains supplementary material available at 10.1007/s10693-022-00378-z.

## Introduction

In the aftermath of the last global financial crisis, policymakers recognised the need for macroprudential policy tools (Arnold et al. 2012, Claessens et al., 2010; Claessens, 2015). Excessive credit cycles have often resulted in financial instabilities with substantial welfare costs. The aim of macroprudential policies is to protect the banking sector and the broader macroeconomy from the destabilising effects of excessive credit growth. In this paper, I provide evidence from two macroprudential measures implemented in Switzerland, namely, a cap on high loan-to-value (LTV) mortgages and a countercyclical capital buffer (CCyB). I find that these measures reduced LTV risks and mortgage growth rates without an increase in risks in other dimensions such as affordability risks, deviating more often from bank internal lending standards or issuing more non-mortgage credit to firms.

Switzerland is an interesting country to study the effects of macroprudential measures for three reasons. First, it was the first country to activate the CCyB in 2013, which was justified by concerns about imbalances in the domestic mortgage and real estate market (SNB 2014).^1^ The CCyB was implemented as a key macroprudential instrument in the Basel III capital regulation (BCBS, 2017). Second, Switzerland is the only country with a *sectoral* CCyB, where the Swiss CCyB targets the mortgage market. A sectoral application of the CCyB has the potential advantages of having a more direct impact on the area of concern, stronger signalling power and smaller effects on the wider economy than the broad-based Basel III CCyB. Such sectoral tools may be useful because lending booms are often confined to a certain sector, such as the mortgage market (BCBS, 2018). Third, Switzerland belongs to the rare number of advanced economies with a large financial sector that implemented an LTV cap in mid-2012, a few months before the CCyB activation in February 2013. Thus, it serves as a case study to analyse the interaction of macroprudential tools.

While both the LTV cap and the sectoral CCyB share a similar objective, namely, to lean against the build-up of systemic risks in the mortgage market, their transmission mechanism is different. The LTV cap targets the LTV distribution of new loans. Mortgage borrowers have to make a down-payment of at least 10% “hard” equity (meaning that the money cannot come from their pension account) when financing a new property. However, it is not a binding cap, i.e., banks can choose to issue mortgages with LTVs over 90% at the cost of holding more capital.

The CCyB is an additional capital requirement on all outstanding mortgage loans, but it does not directly restrict any mortgage characteristics. The CCyB requires banks to build-up capital according to their residential mortgage related risk-weighted assets (RWA). Banks can react to this additional capital requirement in different ways; i.e., they can increase the level of capital (the numerator of the capital ratio) and/or they can shrink their RWA (the denominator of the capital ratio). The former directly increases the loss-absorbing capacity of the banking sector. The latter leans against the build-up of imbalances through two transmission channels. In the first channel, banks reduce mortgage lending because the cost of providing residential mortgages relative to other types of credit increases. In particular, if the banks’ capital situation is tight, imposing additional requirements on already capital constrained banks limits their ability to provide residential mortgages (Drehmann and Gambacorta, 2012). In the second channel, banks reduce their residential mortgage related RWAs by reducing the risks associated with residential mortgage lending. In particular, banks reduce their LTV ratios because they are the key determinants for mortgage related RWAs in the capital regulation.

Because the macroprudential measures were implemented almost contemporaneously and had similar intended effects, disentangling their effects is not straightforward. In this paper, I exploit differences in timing and between banks to estimate the policies’ respective effects. I use a difference-in-differences (DiD) estimator and simultaneously include the different measures. The CCyB treatment group consists of capital constrained banks with a high share of residential mortgage related risk-weighted assets. The LTV treatment group consists of banks that issued a substantial share of their new mortgages with an LTV of over 90% before the cap was introduced. The DiD implementation ensures that my results regarding the CCyB are not driven by the LTV cap implemented beforehand. Moreover, I control for bank and time fixed effects to rule out a potential bias due to unobservable bank characteristics or economic conditions.

My analysis exploits a rich bank panel dataset containing various outcome variables of interest. Among them are credit risk indicators (measured by the share of high LTV and high loan-to-income (LTI) mortgages, exceptions to bank’s lending policies (EtP), mortgage growth rates, and the growth rates of other loans. My sample spans 24 quarters (2011Q2-2017Q1) for the LTV and LTI risks and 34 quarters (2008Q4-2017Q1) for the credit growth rates. It includes the 25 largest mortgage banks covering almost 90% of the Swiss mortgage market. These banks report data to the Swiss National Bank through quantitative surveys. I combine the data on their risk indicators with balance sheet and supervisory data.

My results are that banks more exposed to the LTV cap and the CCyB reduced their high LTV mortgages compared to banks less exposed. The measures affected different parts of the upper LTV distribution, as follows: the LTV cap of 90% reduced new mortgages with LTV ratios over 90%; the CCyB reduced new mortgages with LTV ratios between 80% and 90% at the expense of an increase in new mortgages with LTV ratios between 66% and 80%. The banks affected by the CCyB also reduced their mortgage growth rates. These results suggest that the macroprudential measures exhibited the intended effects. The banks chose to comply with the LTV cap instead of issuing mortgages above the cap at the cost of holding more capital (which would have been possible according to regulation but was not desired by regulators). The CCyB contributed to lean against the build-up of risks; i.e., the banks affected by the CCyB reduced mortgage growth and new mortgages with LTV ratios of more than 80% (which receive a higher risk-weight according to the standardised approach of the capital regulation). At the same time, I do not find evidence that the measures had any unintended effects. Compared to the banks in the control group, the treated banks did not increase their LTI risks, EtP mortgages or non-mortgage lending as a compensation. In the banking system, however, the measures did not prevent the aggregate LTI risks from increasing.

This paper adds to the growing literature on the effectiveness of macroprudential instruments. Most of the existing literature focuses on LTV restrictions, which have primarily been implemented in emerging market economies. These studies suggest that LTV caps are effective in reducing mortgage and house price growth (see Acharya et al. (2021), Allen et al., 2020; Tzur-Ilan (2020); Kinghan et al., 2019; Akinci and Olmstead-Rumsey, 2018; Cerutti et al., 2017; Igan and Kang, 2011; Lim et al., 2011; Morgan et al., 2019; and Wong et al., 2011).

For the *sectoral CCyB in Switzerland*, two other studies exist. Basten (2020) – building on Basten and Koch (2015) – uses data from an online platform to examine how the CCyB affects mortgage pricing. He finds that banks with low capital cushions and high mortgage/asset ratios raise their mortgage rates relatively more than their competitors, but he does not find evidence that these banks raise prices more for high LTV loans. Auer and Ongena (2019) study whether the CCyB affects corporate loans that are not subject to the CCyB. They find that banks with high shares of residential mortgage related RWAs over assets report more corporate loans and increase their interest rates and commissions. They conclude that the sectoral CCyB has caused extra lending in another adjacent sector. When analysing the possible reasons for the differences between my results and the results of the two other studies, I find that the differences in the outcome measurement and treatment definition are relevant, as further discussed in Section 4.5.

This paper advances the literature in three respects. First, I disentangle the effects of the LTV cap and the CCyB. In contrast, the other two studies on the Swiss CCyB do not mention the LTV cap that was implemented in July 2012 with a five months transition period. Given that the CCyB was activated in February 2013 and some banks were affected by both measures, it is important to know how much of the effects should be assigned to each measure. Second, I have a more comprehensive data set. I observe the actual mortgage characteristics of signed mortgage contracts for a longer sample period and a larger sample. Moreover, I estimate the effects for a broader set of outcome variables, i.e., growth rates of mortgages and non-mortgage loans and different credit risk indicators of new mortgage loans. The credit risk indicators are most relevant because credit growth alone may mask important variations in the riskiness of new lending. Third, I refine the measurement of the banks’ exposure to the policy. I show that it is crucial to set the additional capital requirement due to the CCyB in proportion to the bank’s existing excess capital. Out of two banks with the same mortgage/assets or residential mortgages RWA/asset ratios, the more capital constrained bank is more affected by the CCyB.

The remainder of this paper is structured as follows. The next section provides some background on the economic environment and the different macroprudential measures in Switzerland. Section 3 describes the conceptual framework, the data and the empirical strategy. Section 4 provides the results, while the last section concludes.

## Background

### Financial stability risks

As a reaction to the last global financial crisis, the Swiss National Bank (SNB) significantly lowered its target range for the three-month Libor in autumn 2008, introducing a prolonged phase of exceptionally low interest rates. As a consequence, mortgage rates dropped substantially. For instance, the interest rate of a popular mortgage product in Switzerland, namely, a mortgage loan with a fixed interest rate for five years maturity, declined from 3.9% in September 2008 to 1.4% in September 2012. The persistently low interest rates induced an increase in mortgage growth rates (from 3.5% in September 2008 to approximately 5% five years later). Moreover, transaction prices for residential real estate rapidly rose in the same period (by 15% for single family houses and almost 30% for apartments).

The Swiss banking sector is especially exposed to risks in the mortgage sector. Mortgages constitute the most important asset for many Swiss banks; at a typical bank in my sample, 70% of the balance sheet are mortgages.^2^ Moreover, the mortgage risk remains on the banks’ balance sheets, as there is no securitisation.^3^

To address the risks in the mortgage and real estate markets, Swiss policymakers have implemented different macroprudential measures. According to their assessment, a single measure would not have been sufficient. The most important measures were an LTV cap in 2012 and the activation and increase of a countercyclical capital buffer in 2013 and 2014. Different institutions were involved in the design and implementation of macroprudential measures. All of them shared the concern over a build-up of risks in the mortgage markets, but had different roles and responsibilities. The Swiss Financial Service Authority (FINMA) has implemented the LTV cap within the banker’s self-regulation. The SNB proposed the activation of the CCyB to the Federal Council after consultation with FINMA. Then, the Federal Council decided to follow the SNB’s proposal. Next, I explain the different measures in more detail.

### Loan-To-Value ratio cap

In 2012, the FINMA introduced an LTV cap within the self-regulation of the Swiss Bankers’ Association. It required a down-payment of at least 10% “hard” equity for financing a new residential property. “Hard” equity means own funds without pension savings. Thus, mortgage borrowers were no longer allowed to finance their down-payment by relying exclusively on their pension savings. Using pension savings as part of a down-payment is a widespread practice in Switzerland (Seiler, 2013) and is explicitly supported by Swiss law.

However, the LTV cap was not a strict ban. Because of competition concerns, banks were given the choice between issuing a new mortgage loan (i) with an LTV less than 90% within the normal risk-weighting scheme or (ii) with an LTV above 90% but applying a 100% risk-weight for the entire mortgage loan. The 100% risk-weight is a considerable increase compared to the average risk-weight of approximately 40% for a typical residential mortgage loan.^4^ Thus, banks have strong incentives to issue mortgages below the cap if they want to contain their RWA; nevertheless, other considerations (e.g., winning new customers) could still lead them to issue loans with LTV ratios above 90%.

The LTV cap was announced in June and became effective in July. Banks were given a transition period of five months, i.e., until the end of November 2012, when they could issue mortgages with LTV ratios over 90% without applying a 100% risk-weight. In my baseline regressions, I assume the third quarter of 2012 as the LTV cap’s beginning, because it corresponds to the quarter, in which most of the banks have revised guidelines according to a qualitative survey. A few banks have adjusted their behaviour already in the second quarter 2012, but my results are robust with respect to this anticipation, as I will discuss in more detail later.

### Countercyclical Capital Buffer

The CCyB has been part of the international macroprudential toolkit since 2016. It requires banks to accumulate capital as imbalances in the credit market develop (Basel III, 2011). Depending on the degree of systemic risk, a countercyclical capital requirement between zero and 2.5% of risk-weighted assets is put in place. It is increased when the systemic risks rise; it is released when the systemic risks materialise or dissipate. According to Basel III, the CCyB is applied on a broad basis, i.e., to total risk-weighted assets (RWA).

In Switzerland, the CCyB targets exposures in the residential mortgage sector, i.e., temporarily raises capital requirements for mortgage loans on residential property. The Swiss authorities chose this sectoral variant of the CCyB (although the broad CCyB has been available to them since mid-2012), because they were concerned about the risks in the mortgage market. The intended effects of the CCyB are twofold, as follows: (i) to increase the resilience of the banking sector by increasing its loss-absorbing capacity, and (ii) to lean against the build-up of excessive credit growth by limiting the potential for lending, given the current capital available (SNB 2014).^5^

The Federal Counsil announced the activation of the CCyB in February 2013, following a proposal by the SNB and consultation with the FINMA. The authorities kept the decision making phase confidential. Banks could neither foretell its activation, its level nor its application to residential mortgage loans. At its activation, it was set to a level of 1% of the residential mortgage associated risk-weighted position, with a deadline of compliance at end of September 2013. In my baseline regressions, I set the quarter of the announcement (2013Q1) as the beginning of the CCyB assuming that banks will start the lengthy adjustment process immediately. In robustness test, I will show that banks were indeed surprised by the announcement and adjusted their LTVs right away. In 2014, the level of the CCyB was increased to 2%. The increase was announced in January 2014, with a compliance deadline at the end of June 2014. In March 2020, the CCyB was released to support banks in their key role as lenders in the coronavirus crisis.

The CCyB increased the overall capital requirements in the banking system somewhat. For the 25 largest mortgage banks in my sample, the additional required capital due to the CCyB activation was 3% of their total minimum capital requirement. There was considerable heterogeneity among these banks; i.e., the CCyB ratio required capital over the total minimum required capital, which ranged from 1% to 8%, depending on the relative importance of residential mortgage related RWA. Moreover, these banks differed considerably with respect to their capital buffers; i.e., some banks had almost no excess capital relative to regulatory requirements, while many had a capital/RWA ratio more than four percentage points higher than their bank specific target ratio. I will exploit the heterogeneity between the banks to identify the effects of the CCyB.

### Other policy measures

Swiss policymakers also implemented other policy measures between 2012 and 2014. The most important ones are summarised in Table 1. One policy measure was an increase in the risk-weight for residential mortgage lending; i.e., the risk-weight for the tranche exceeding the 80% LTV ratio was increased from 75% to 100%. Because the higher risk-weight only applies to tranches, i.e., only to the part of the mortgage that actually exceeds the threshold, and because most of the outstanding mortgages have an LTV below the 80% threshold, I expect its effect to be small. In a robustness test, I will show that my results on the coinciding CCyB activation are not confounded by the increase in risk-weight.

**Table 1:** Overview of the policy measures implemented

**Quarter**	**Measure**	**Description**	**announced**	**effective**
2012Q3	1st revision of the self-regulation	10% “hard” equity down-payment	1.6.2012	1.7.2012, with a five-month transition period
2013Q1	Increased risk-weight	for loan tranche exceeding LTV of 80%	1.6.2012	1.1.2013
2013Q1	Activation of the CCyB	1% of residential mortgage related RWA	13.2.2013	30.9.2013
2014Q1	Increase in the CCyB	2% of residential mortgage related RWA	23.1.2014	30.6.2014
2014Q3	2nd revision of the self-regulation	Linear and yearly amortisation requirement	24.6.2014	1.7.2014, with a five-month transition period

Another policy measure was an amortisation requirement introduced in the revised self-regulation in 2014.^6^ New mortgage borrowers were required to amortise their loans to an LTV of 2/3 within the next 15 years, where they should amortise yearly and linearly. Because I do not have the data to assess the effect of this requirement, I only control for it by including a time dummy variable in my estimation.

## Methods and data

### Conceptual framework

Both the LTV cap and the CCyB share a similar broad objective, namely, to lean against an accumulation of systemic risk in the mortgage market. However, their transmission channels are different. The LTV cap targets the upper part of the LTV distribution of new mortgages. However, banks can choose whether they reduce new mortgages with LTV ratios over 90% or issue them at higher capital cost. The CCyB applies to outstanding and new mortgages and directly increases their capital costs. The banks can react to the associated capital costs in different ways. I discuss my hypotheses regarding the effects of the two measures in succession.

Regarding the LTV cap, an effect on the right tail of the LTV distribution is most likely. Banks will reduce new mortgages with LTV ratios over 90% at the threshold to avoid much higher costs in terms of capital. However, further away from the threshold, the bank’s optimal choice might be different. In particular, acquiring new customers and expanding profit margins might outweigh the increased capital cost. In addition to the banks’ decisions, the borrowing decisions of first time buyers are relevant, because they are the group that is most likely to take out a mortgage with an LTV over 90%. Depending on their time preferences, financially constrained first time buyers can either buy cheaper houses or postpone their purchase until their savings are sufficient. Empirical evidence on their reaction is mixed. In Israel, borrowers bought cheaper houses, farther from central business districts and in lower social economic neighbourhoods (Tzur-Ilan, 2020). In Canada, first-time borrowers made larger down-payments as a fraction of their income, while house prices increased (Allen et al., 2020). In Ireland, first-time borrowers increased their down-payments, with no change in the house price paid (Kinghan et al., 2019).

The CCyB acts as a lending restriction to capital constrained banks, but it does not directly impose any restrictions on mortgage growth or characteristics. Following Gambacorta and Mistrulli (2004), banks hold capital in excess of capital requirements, either because they face capital adjustment costs or they want to convey positive information regarding their economic value. Banks are different with respect to their chosen excess capital; some banks find it optimal to operate close to the regulatory intervention threshold, while others operate far away from it. Some possible reasons for this bank heterogeneity are the differences in equity costs, private costs of bankruptcy or risk aversion.

The activation of or an increase in the CCyB implies a higher regulatory capital requirement and, hence, a shock to the chosen excess capital. Capital constrained banks with a high share of residential mortgages in their portfolio are hit harder by the additional capital requirement. Thus, I expect to observe a reaction by those banks. Other banks hardly need to react given their large amounts of excess capital and the temporary nature of the CCyB requirement.

In principle, banks can choose different strategies to restore their chosen excess capital. They can either increase their capital and/or reduce their RWA. Because issuing equity is costly and lengthy, banks are likely to reduce their RWA. This can be achieved by cutting back the mortgage lending or risks determining the risk-weighting of mortgages.

In addition to reducing their mortgage volumes, I expect banks to reduce their RWA by adjusting their LTV distributions. The reason is that the LTV ratio is a key determinant for mortgage related risk-weighted assets under both the standardised approach (SA) and the internal model based (IRB) approach to capital regulation.^7^ As 22 banks in my sample apply the SA, I expect that the LTV thresholds used in this approach (66% and 80%) are relevant (see footnote 4).

However, both measures could also have unintended consequences (Jimenez et al., 2017). They put pressure on the bank’s profitability opportunities. To maintain their profitability, the banks might want to increase risks in other dimensions to compensate. In particular, they could increase their LTI risks (which are not regulated under the SA) or switch to riskier types of credit than mortgage loans. For instance, Norwegian and UK banks reacted to an increased capital requirement by originating riskier loans (Juelsrud and Wold, 2020; Uluc and Wieladek, 2018), while Irish banks reacted to LTV and LTI limits at home by increasing their risks abroad (McCann and O’Toole, 2019) and increased their risk taking in asset classes not targeted by the lending limits such as corporate lending and high-yield securities (Acharya et al., 2021).

Finally, spillover effects could arise within the banking system from treated to control banks or outside the banking system from regulated banks to other sources of finance, e.g., insurers or pension funds, (Aiyar et al., 2014; Kim et al., 2018) or from regulated banks at home to bank lending abroad (Buch and Goldberg, 2017). The market share of non-bank mortgages accounts for only approximately 5% and has been stable in recent years. Hence, I am more concerned about spillover effects within the banking system than leakages to non-banks. With the exception of the two big banks, the Swiss banks in my sample have little international exposure, thus limiting the scope of spillovers for lending abroad.^8^

### Data and descriptive statistics

My sample consists of the 25 largest mortgage banks in Switzerland. Their mortgages account for almost 90% of the mortgage market. These banks report information on new residential mortgage loans to private households in a quarterly survey conducted by the SNB (2011Q2-2017Q1). Data are on a bank-mortgage portfolio level: banks report the LTV and LTI distribution of their residential mortgage portfolio. The respective numerators are the credit limits of the new mortgage loans. The value in the LTV denominator is the market value of the pledged property.^9^ The income in the LTI denominator is the borrower’s net employment or pension income. The relationship between LTV and LTI is a priori not clear. It could be positive because both ratios share the same numerator or negative because banks balance both risk dimensions. Evidence from a loan-level survey (that starts in 2017) shows a globally positive correlation, but also that for a given LTV ratio the LTI ratio may take a wide range of values. Moreover, the local linear regression coefficient varies depending on the LTV ratio; it is positive for an LTV ratio between 0 and 60% and over 80%, close to zero for an LTV between 60% and 75% and slightly negative for an LTV ratio between 75% and 80% (see Fig. A.1 in the Appendix). As a third risk indicator, they report the share of new residential mortgage loans that does not comply with banks’ lending standards (also known as exception-to-policy (EtP) loans).

**Fig. 1:**
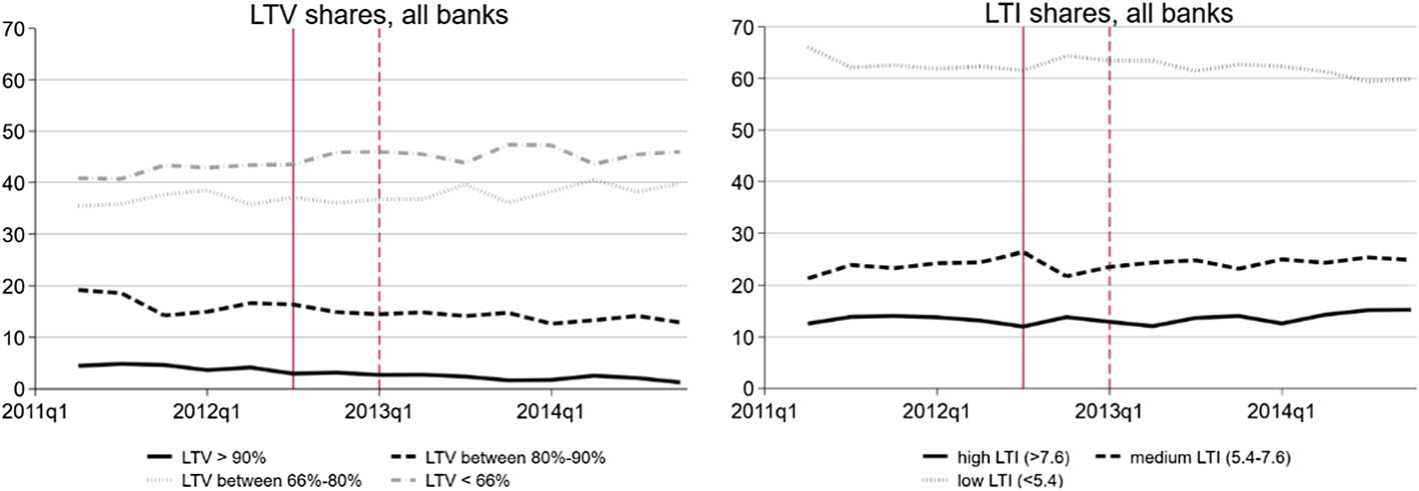
Average share of new mortgages by LTV (left) and LTI (right) bucket (2011Q2-2014Q4). The vertical lines indicate the implementation of the LTV cap (2012Q3) and the activation of the CCyB (2013Q1)

As first set of outcome variables, I examine the shares of new residential mortgages with high LTV ratios because these are the high risk mortgages. For instance, banks that provide many mortgages with LTV ratios of over 90% run the risk of having a high proportion of underwater mortgages in their portfolio should real estate prices fall by 10%. I define additional LTV buckets according to the LTV thresholds under the standardised approach, i.e., 66% and 80% (see footnote 4). The overall LTV distribution was shifted to the left towards lower risk between 2011 and 2015 (see left panel in Fig. 1). The share of new mortgages with LTV ratios of more than 90% declined from 5% to 1%, the share with LTV ratios between 80% and 90% declined from 19% to 13%, while the shares with LTVs between 66% and 80% increased from 35% to 40% and the share with LTV less than 66% increased from 41% to 46%. There is neither a sharp LTV decline after the LTV implementation nor after the CCyB activation observable in the aggregate sample. Applying microeconometric techniques will reveal that it were the banks affected by these measures that reduced their high LTV shares.

As second set of outcome variables, I analyse the shares of new mortgages with high LTI ratios indicating high affordability risks. I select different LTI thresholds in order to analyse different parts of the LTI distribution. I consider an LTI ratio of more than 7.6 as very high, one with LTI between 5.4 and 7.6 as medium and one with LTI below 5.4 as low. These thresholds result from a “golden rule” according to which interest, amortisation and maintenance cost should not exceed one-third of cost of gross wage income.^10^ In the overall sample, the shares of mortgages with high (from 13% to 15%) and medium (from 21% to 25%) increased between 2011 and 2015 (see right panel in Fig. 1). This increase is not driven by banks affected by the two macroprudential measures, as I will show later.

As a third risk dimension, I look at the share of new mortgages that does not comply with banks’ lending standards (also known as exception-to-policy (EtP) loans). Banks use this flag to monitor risks in their mortgage portfolios. Banks’ credit policies vary in many dimensions. Among them are the calculation of sustainable income (treatment of second income, bonuses, capital income, other financial assets, and costs for other credits, expected retirement), assumed credit costs, amortisation rules, accepted collateral and so forth. Due to this variation, it is difficult to collect standardised information on these separate items. However, I can observe whether banks classify more mortgage loans as “exception” to its own policy. In my sample, this share was on average 23% (see Fig. A.2 in Online Appendix).

As a fourth set of outcome variables, I analyse yearly growth rates of lending volumes. In particular, I look at the total mortgage volume outstanding and then separate at mortgages for private households and firms. Most of mortgages by private household and about half of mortgages by firms is residential (and hence potentially affected by the CCyB). About 5% of the mortgage stock are new residential mortgages to privates that were potentially affected by the LTV cap. Finally, I look at the yearly growth rate of other (non-mortgage) loans to firms to analyse whether there have been any unintended spillover effects. The average growth rates exhibit cyclical pattern and declined between 2011 and 2015 (see Fig. A.2 in Online Appendix).

### Empirical strategy

In this application, selection into treatment is not at random. Banks extending high LTV mortgages were more likely to be affected by the LTV cap. Similarly, mortgage specialised and capital constrained banks were more likely to be affected by the CCyB. I use the difference-in-differences (DiD) to estimate the causal effects of the two macroprudential measures policy tools. In particular, I estimate different regressions of the following form.

1 $${y}_{it}={\beta}_1{LTVexp}_i\ast {T}_{2012Q3}+{\beta}_2{CCyBexp}_i\ast {T}_{2013Q1}+{\alpha}_i+{\gamma}_t+{\epsilon}_{it}$$

In this equation, *y*_*it*_is an outcome variable of interest, for instance the share of new mortgages with high LTV or LTI ratios. *LTVexp*_*i*_ and *CCyBexp*_*i*_ measure the treatment intensities, i.e. how much a particular bank was affected by the respective macroprudential measure. In robustness tests, I will also use different dummy indicators dividing banks into treatment and control groups. The time dummies *T*_*2012Q3*_*, T*_*2013Q1*_ are 0 before and turn 1 in the quarter when the respective measure is implemented. In robustness tests, I will also estimate dynamic DiD by replacing the two time dummies with quarterly time dummies. Moreover, I control for time-invariant bank characteristics and common shocks by including bank *α*_*i*_ and quarter *γ*_*t*_ fixed effects. In robustness tests, I will also control for time-varying bank characteristics and bank’s cantonal market shares. The main coefficients of interest *β*_*1*_ and *β*_*2*_ measure the differential effect of being exposed to the respective macroprudential measure in the treatment period. In the presence of non-random selection into treatment, the DiD identifies the counterfactual outcome under two assumptions. First, banks do not change their behaviour in anticipation of treatment. Second, the average outcomes in both groups are subject to a common time trend that is conditional on the covariates*.* I will show that both assumptions are by and large satisfied.

#### Definition of Treatment

Assuming that banks with more mortgages with LTV over 90% before treatment were more affected by the LTV cap, I measure each bank’s exposure to the LTV cap as:

$${LTVexp}_i=\frac{1}{4}\sum_{t=2011Q2}^{2012Q2}\frac{new\ mortgages\ with\ LTV\ over\ 90{\%}_{it}}{new\ {mortgages}_{it}},$$

i.e. the average share of new mortgages with LTV ratios over 90% between 2011Q2 and 2012Q2. This outcome definition is clearly predetermined with respect to the LTV cap introduced in July 2012. Table A.1 in the Online Appendix shows that a few banks did not issue any mortgages with LTV of more than 90%, while at some banks almost 10 % of new mortgages had an LTV ratio of more than 90% before the self-regulation was announced. Since the median is close to 5% and the distribution is symmetric around the median, I use 5% as cut off value in my baseline dummy specification. Thus, in the treatment group (*LTV*_*dum*_) are the twelve banks whose average share of new mortgages with LTV over 90% was higher than 5% before 2012Q3. Ten out of these twelve banks also report to have adjusted their lending standards due to the revised self-regulation in a qualitative survey among senior loan officers. I will also test different thresholds for the dummy definition.

To measure how much a bank was affected by the activation of the CCyB, I rely on supervisory capital data. I measure each bank’s exposure to the CCyB activation as:

$${CCyB exp}_i=\frac{{CCyB\ required\ capital}_{i,2012Q4}}{{\left( actual\ capital- target\ capital\right)}_{i,2012Q4}}\kern1.25em ,$$

i.e. the required CCyB capital relative to the bank’s excess capital. To avoid endogeneity problems, I use the predetermined excess capital end-2012.^11^

For illustration purposes, let us consider four stylised Swiss banks that are different with respect to their excess capital and residential mortgage risk taking. They have 30 CHF bn assets, 21 bn residential mortgages, 15 bn RWA and their regulatory target capital/RWA is 12. The two well capitalised banks have 2.46 bn in actual capital (corresponding to a capital/RWA of 16.4); the two tightly capitalised banks have 1.86 bn in actual capital (corresponding to a capital/RWA of 12.4). Two of the banks have relatively low-risk mortgage portfolios, with residential mortgage RWA (RRWA) values of 7.5 bn each (corresponding to an average risk-weight of 0.36), while two others have riskier mortgages, with RRWA values of 10.5 bn each (corresponding to an average risk-weight of 0.50).

Table 2 shows each bank’s CCyB treatment exposure as the ratio of CCyB required capital over excess capital, where the CCyB is set to 1% of the RRWA. This CCyB treatment intensity variable implies a stronger CCyB effect for banks (i) with higher residential mortgage risk and (ii) with smaller excess capital.

**Table 2:** CCyB treatment intensity for four stylised Swiss banks (CCyB: 1% of RRWA)

	RRWA in CHF bn	RRWA/ mortgages	Actual capital/ RWA	CCyB required capital in CHF bn	excess capital in CHF bn	CCyB exposure
well capitalised, low mortgage risk	7.5	0.36	16.4	0.075	0.66	0.11
well capitalised, high mortgage risk	10.5	0.50	16.4	0.105	0.66	0.16
tightly capitalised, low mortgage risk	7.5	0.36	12.4	0.075	0.06	1.25
tightly capitalised, high mortgage risk	10.5	0.50	12.4	0.105	0.06	1.75

The actual distribution of the CCyB treatment exposure variable is shown in Table A.1 in the Appendix. There are many banks with a low CCyB treatment exposure. At the median bank it was 0.12 (similar to the numbers for the two well capitalised stylised banks). Because the CCyB treatment exposure is positively skewed with only a few banks having a high intensity, I use the 80th percentile (0.4) in my baseline dummy specification (*CCyB*_*dum*_). Accordingly, there are four banks in the treatment group reflecting the fact that most of the banks in my sample are well capitalised and have low risk-weights for residential mortgages (the average RRWA/mortgage is 0.34). In robustness checks, I will also consider different thresholds for the dummy specification.

I also calculate the treatment intensity for the CCyB increase in 2014. However, the treatment exposure variables are highly correlated with each other (coefficient of 0.8) because only one year passed between the activation and the increase. Due to this multicollinearity, I do not have sufficient statistical power to separately identify the effects of the CCyB increase.

#### Statistical Inference

My sample size (N = 25, T = 24) is given by the coverage of the mortgage survey that is used to define the treatment and control groups. A small number of N will reduce the statistical power. To obtain the correct inference, I take into account that I have only a few clusters. In particular, I use a wild cluster bootstrap procedure suggested by Cameron, Gelbach, and Miller (2008). It has been often been applied in cases where the number of clusters was smaller than the 25 (banks, in my case) (see Behncke, 2012; Brown et al., 2009).

## Results

### LTV cap and CCyB exposure

The differential effects of the two macroprudential measures are shown in Table 3. In order to compare the magnitudes of their effects, I have standardised the *LTV*_*exp*_ and *CCyB*_exp_ so that they have mean zero and standard deviation one. The interaction in the first row and first column shows that banks with a one-standard-deviation higher LTV treatment exposure differentially reduced their share of new mortgages with LTV over 90% by 2.3 percentage points after the LTV cap was introduced. Given that this share was 2.55% on average in my sample, this is a sizeable effect. Thus, the introduction of the LTV cap of 90% was effective in reducing high LTVs. Banks chose to reduce LTV risks (as intended by policymakers) instead of maintaining them at the cost of higher capital (which would have been possible according to the regulation).

**Table 3:** DiD regression results of LTV and CCyB treatment intensities. All regressions control for bank and quarter time fixed effects. *LTV*_*exp*_ and *CCyB*_*exp*_ measure the standardised treatment intensities of the LTV cap and the CCyB activation. *T*_*2012Q3*_ resp. *T*_*2013Q1*_ are time dummies equal to 1 from 2012Q3 resp. 2013Q1 onward. EtP denotes Exception-to-Policy mortgages

	**Mortgage share with LTV**				**… with LTI**		**… with**	**Mortgage growth**			**credit**
	**>90%**	**80–90%**	**66–80%**	**<66%**	**>7.6**	**5.4–7.6**	**EtP**	**total**	**private**	**firms**	**firms**
**LTV**_exp_*T_**2012Q3**_	−2.30***	−0.49	1.59	1.20	−1.22	−0.70	−3.81	−0.51	−0.72	−0.01	0.47
	(0.30)	(1.14)	(1.28)	(0.73)	(1.16)	(0.74)	(2.42)	(0.41)	(0.44)	(0.63)	(0.61)
**CCyB**_exp_* T_**2013Q1**_	−0.33***	−1.55***	1.95***	−0.07	−0.04	−1.08	−0.19	−0.37*	−0.39	−0.27	0.11
	(0.07)	(0.25)	(0.53)	(0.42)	(0.33)	(−0.69)	(0.70)	(0.20)	(0.24)	(0.22)	(0.21)
Observations	600	600	600	600	575	575	600	850	850	850	850
R-squared	0.56	0.58	0.41	0.66	0.61	0.54	0.60	0.58	0.60	0.50	0.38
Mean (dep. Var.)	2.55	14.52	38.30	44.63	14.88	24.59	21.25	4.61	4.61	4.81	4.09
Std (dep. Var.)	3.09	6.63	6.80	7.73	9.42	7.26	13.58	2.41	2.75	5.08	5.92

Standard errors are determined by a wild cluster bootstrap with ***,**,* denoting significance at the 1%, 5% and 10% levels, respectively

Except from the upper part, the other parts of the LTV distribution were not affected at conventional significance levels. Thus, there was no strong substitution from the upper LTV part to a certain bucket in the lower part.

In addition, I do not find any statistically significant effects on mortgage growth rates. Thus, the LTV cap reduced the risk density of mortgages without contracting mortgage lending. The absence of substitution and mortgage growth effects together might indicate that borrowers had sufficient funds to provide the “hard” equity but would have preferred to use their pension savings. A widespread substitution to cheaper houses or postponement of house purchases would have resulted in declining mortgage growth rates.

I do not find any other statistically significant effects related to banks’ differential exposure to the LTV cap. The banks did not increase their LTI risks or their share of EtP mortgages to compensate for the reduced LTV risk. They also did not significantly change other types of lending to firms. The LTV risk density of new mortgages was reduced without any unintended consequences on observable risk dimensions.

Next, I discuss the differential effects on banks more exposed to the CCyB activation (shown in the second row of Table 3). The LTV distribution was significantly affected, as follows: mortgages with LTVs of more than 90% and between 80% and 90% declined, while mortgages with LTVs between 66% and 80% increased.^12^ Thus, the LTV distribution was compressed. The effects are quantitatively important relative to the sample average (shown in the penultimate row): per one standard deviation higher exposure to the CCyB, banks reduced their share in the above 80% LTV bucket by 1.88 (0.33 + 1.55) percentage points and increased their share in the 66%–80% LTV bucket by 1.95 percentage points after the CCyB activation. Through this substitution, they could reduce their residential RWA because below the 80% threshold the risk-weight is smaller in the standardised approach to capital regulation (see footnote 4).

Moreover, banks more affected by the CCyB reduced their mortgage growth by 0.37 percentage points. This effect, though, is only significant on the 10% significance level because of effect heterogeneity, as I will discuss below.

As for the LTV cap, I do not find any evidence that banks increased risk in any other observable dimension in order to compensate the higher capital costs of issuing mortgages. Thus, both macroprudential measures appear to have contributed to financial stability.

### Treatment dummy specifications

In a first robustness check, I estimate a specification of the DiD regression in which I replace the treatment intensities by *LTV*_*dum*_ and *CCyB*_*dum*_. The dummy specification has the advantage that the coefficients are easy to interpret (average impact on treatment group relative to control group) and are less sensitive to outliers. But the researcher has to decide on an appropriate cut off dividing banks into a treatment and control group. As discussed in Section 3.3, I select the cut offs guided by the distribution of the treatment intensities and test different cut offs.

For the LTV cap, the coefficients in the dummy specification remain qualitatively similar, as Table 4 shows. The share of mortgages with LTV ratios of more than 90% is 4.4 percentage points smaller for the twelve banks in the LTV treatment group compared to the control group after the LTV cap has been implemented. Compared to the LTV exposure specification, the absolute size of coefficient increases: the twelve banks in the treatment group reduced their over 90% LTV bucket from 7.1% before to 2.5% after the implementation of the LTV cap, while the 13 banks in the control group only reduced it from 1.8% to 1.7%. The other coefficients are again not statistically significant.

**Table 4:** DiD regression results (dummy specification). All regressions control for bank and quarter time fixed effects. *LTV*_*dum*_ and *CCyB*_*dum*_ measure whether a bank was in the LTV or CCyB treatment group. *T*_*2012Q3*_ resp. *T*_*2013Q1*_ are time dummies equal to 1 from 2012Q3 resp. 2013Q1 onward. EtP denotes Exception-to-Policy mortgages

	**Mortgage share with LTV**				**… with LTI**		**… with**	**Mortgage growth**			**credit**
	**>90%**	**80–90%**	**66–80%**	**<66%**	**>7.6**	**5.4–7.6**	**EtP**	**total**	**private**	**firms**	**firms**
**LTV**_dum_*T_**2012Q3**_	−4.37***	−0.27	2.35	2.28	−0.69	−1.61	−6.70	−0.79	−0.59	−0.81	0.12
	(0.72)	(1.90)	(1.88)	(1.35)	(2.21)	(1.75)	(4.19)	(0.72)	(0.84)	(1.39)	(1.52)
**CCyB**_dum_* T_**2013Q1**_	−0.44	−3.50**	8.88***	−4.95***	−3.54	−0.08	−2.09	−2.04***	−2.53***	−1.29	1.64
	(0.62)	(1.59)	(1.29)	(1.72)	(2.86)	(2.17)	(3.87)	(0.59)	(0.80)	(0.79)	(1.54)
Observations	600	600	600	600	575	575	600	850	850	850	850
R-squared	0.55	0.57	0.44	0.67	0.61	0.53	0.59	0.60	0.62	0.50	0.38
Mean (dep. Var.)	2.55	14.52	38.30	44.63	14.88	24.59	21.25	4.61	4.61	4.81	4.09
Std (dep. Var.)	3.09	6.63	6.80	7.73	9.42	7.26	13.58	2.41	2.75	5.08	5.92

Standard errors are determined by a wild cluster bootstrap with ***,**,* denoting significance at the 1%, 5% and 10% levels, respectively

Similarly, the size of several coefficients increases in the *CCyB*_*dum*_ compared to the *CCyB*_*exp*_ specification. The four banks in the CCyB treatment group reduced their 80%–90% LTV bucket by 3.5 percentage points and increased their 66%–80% LTV bucket by 8.9 percentage points compared to the control group after the CCyB was activated. Both effects are large corresponding to more than 20% of the average in the overall sample. Moreover, the banks in the CCyB treatment group reduced their mortgage growth rates in total and to private households by 2 resp. 2.5 percentage points. The reduction in growth rates is much more substantial in the dummy specification compared to Table 3.

Effect heterogeneity explains why the size of several coefficients increases in the dummy specification. In contrast to the other three banks in the CCyB treatment group, the bank with the highest treatment intensity did not reduce its mortgage growth rates after the CCyB activation, as Table A.2 in the Appendix shows. Since there is no proportional relationship between the CCyB treatment intensity and the reduction in growth rates, the effects are smaller in the CCyB treatment intensity specification. Both specifications generate significant effects on mortgage growth rates if the bank with the highest CCyB treatment intensity is excluded from the sample.^13^

These results are stable with respect to different cut offs in the dummy treatment definition. The Appendix reports results from a rolling group test where different number of banks are included in the respective treatment groups (see Table A.3 and A.4).

### Dynamic treatment effects

**Fig. 2:**
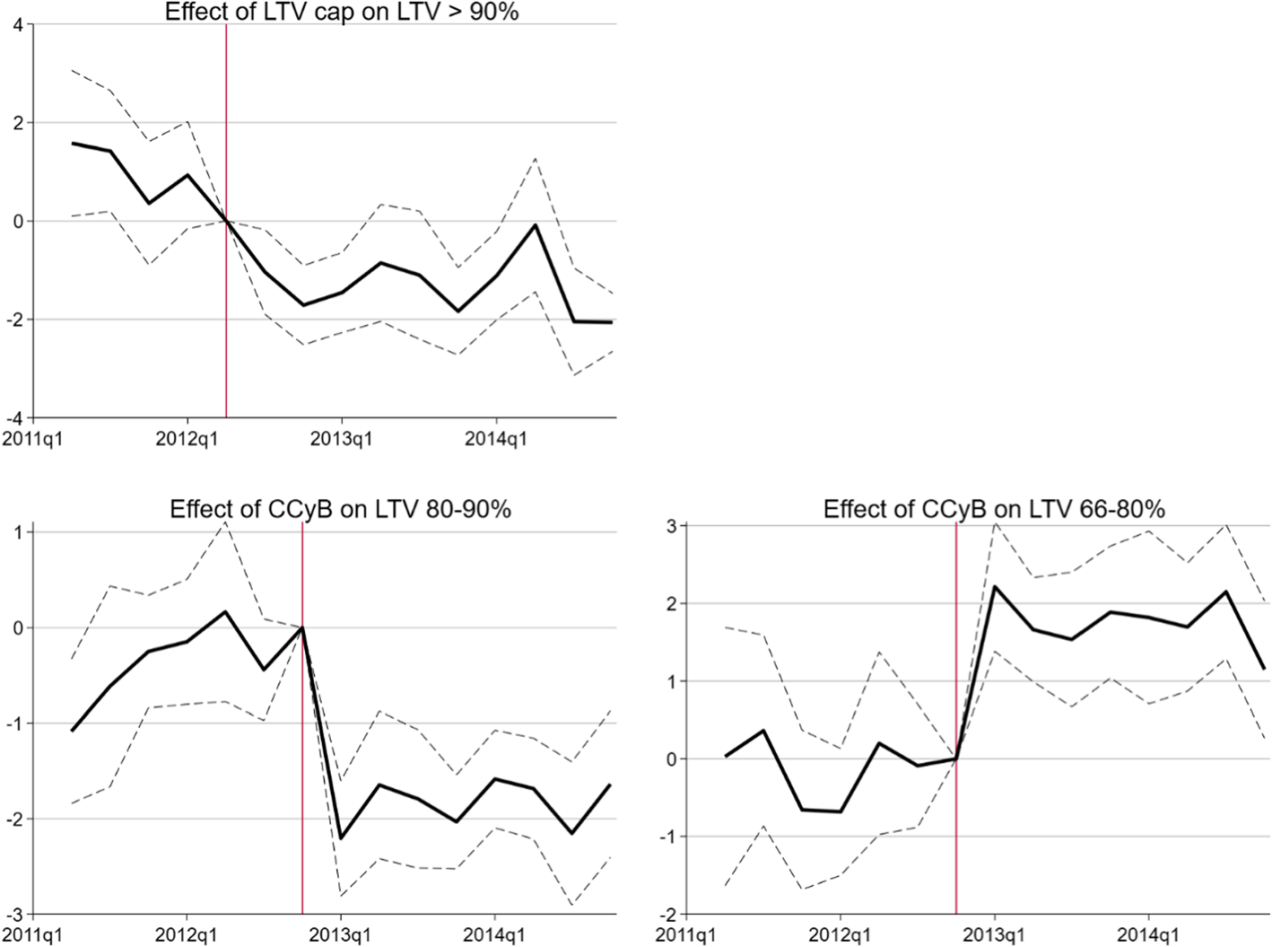
Dynamic DiD plots, by LTV (above) and CCyB (below) treatment intensity. The solid line shows estimated coefficients with quarterly time dummies. The dotted line corresponds to the 95% confidence interval. The vertical lines indicate the quarters *before* the implementation of the LTV cap (2012Q2) in the upper panel and *before* the activation of the CCyB (2012Q4) in the bottom panel

Next, I estimate marginal treatment effects per quarter to analyse the validity of the common trend and no-anticipation assumptions. In particular, I replace the two time dummies with quarterly time dummies setting the quarter before the implementation of the respective measure as reference quarter. Figure 2 shows the results for the outcome variables for which the previous analysis documented significant and robust effects. The results for the other outcome variables are reported in Figs. A.2 and A.3 in the Appendix.

These Figures illustrate the absence of concerning pre-trends. Before the implementation of the respective macroprudential measure, the estimated coefficients are generally close to zero and not statistically significant. After the implementation of the LTV cap, the effect on the share of new mortgages with LTV over 90% turn negative and are significant in most quarters (Fig. 2, upper panel). After the activation of the CCyB, the coefficients on the 80%–90% and 66%–80% bucket become significant with opposing signs (Fig. 2, bottom panel).

To analyse potential anticipation effects, I estimate the baseline regressions with the time dummies shifted a quarter before the treatment in the baseline regression. In these regressions, I find a significant marginal effect of the LTV cap in 2012Q2 and otherwise insignificant coefficients before treatment. This evidence suggests that banks could not foresee the activation and the design of the CCyB, but some banks in the LTV treatment group reduced their high LTV ratios already in the second quarter 2012, in which the LTV cap was announced. Some of these banks were taking part in working groups in which they discussed the revisions of the self-regulation with the FINMA. Despite of the five-month transition period until end-November 2012, they preferred to react immediately, possibly in order to signal compliance to FINMA. Indeed this is confirmed by senior loan officers in the qualitative survey mentioned above. Accordingly, three banks have adjusted their lending standards already in 2012Q2, while seven have adjusted them in 2012Q3.

The anticipation of the LTV cap does not qualitatively affect my results. Running the baseline regression with 2012Q2 instead of 2012Q3 as interaction results in similar coefficients, as Table A.4 in the Appendix shows. While a sudden policy shock without anticipation would have been preferable for my identification strategy, it is reassuring that the coefficients point in the same direction as in my main specification.

### Further robustness checks

In this section, I analyse several potential confounds that one could be concerned about. Altogether the findings suggest that my baseline specifications – in which I simultaneously include both macroprudential measures, bank and quarter fixed effects – generate robust results.^14^

One concern is that various policies were implemented or changed around the same time. For instance, the increase in risk-weights in the standardised approach (SA) to capital regulation was announced in June 2012 and became effective from January 2013 on. Thus, it coincides with the LTV cap announcement in June 2012 and the CCyB activation announcement in February 2013. If all banks in my sample used SA, the quarterly time dummies would completely absorb the increase in risk-weight. However, three banks use the IRB approach and could pose a threat for identification, if they were unbalanced with respect to treatment and control group. To test whether this issue is relevant, I add the interaction IRB bank dummy times a dummy that is equal to one from 2013Q1 onward. Table A.5 in the Appendix shows that the coefficients of interest do not change.

Similarly, I estimate the effects of the LTV cap in a sample that stops before the CCyB activation was announced in order to get a clean impact of the LTV cap alone. The effects remain similar, as Table A.7 in the Appendix shows. The coefficient on the above 90% LTV bucket is with −1.99 somewhat smaller in magnitude due to the reduced sample period and resulting lower statistical power, but remains highly significant.

Another concern is that bank fixed effects, which have been used throughout, may not capture all relevant bank characteristics. If banks’ characteristics changed over time and these changes were correlated to their risk taking, bank fixed effects would not be sufficient. To address this issue, I add controls for time-varying bank characteristics in addition to bank and time fixed effects. I select those time-varying characteristics that are exogenous to the treatment, but correlated with bank’s risk taking appetite. In particular, I include return-on-assets and cost-to-income as measures for bank’s profitability and efficiency. I include deposits over assets, trading assets over assets, commission income over operating income, trading income over operating income to measure changes in bank’s business model. I also include a measure for loan losses and funding costs. The main coefficients of interest are qualitatively similar to the baseline regression, as Table A.7 shows.

Similarly, regional differences between banks could confound the analysis if treated banks were concentrated in certain cantons. In my sample, 17 banks are active in only one or two cantons. To test whether treated banks are concentrated in certain cantons, I calculate treatment intensities per canton by weighting each bank’s respective treatment exposure with its cantonal market share end-2011. Most of the 26 cantons are balanced with respect to the two treatment intensities. One very small canton has a high LTV treatment intensity and a few very small cantons (accounting to 10% of Switzerland’s mortgage market) have a high CCyB treatment intensity. To address this issue, I add each bank’s regional concentration in these cantons interacted with the ex post time dummies in addition to the bank and time fixed effects. Table A.8 shows that results remain robust in this specification. The coefficients of the CCyB treatment intensity even increase in magnitude.

### Comparison to other studies

In this section, I analyse why some of my findings differ from those in the existing work on the CCyB in Switzerland. Specifically, Auer and Ongena (2019) find that the CCyB activation resulted in an increase in commercial loans, while I find insignificant effects on non-mortgage lending to firms. In Basten (2020), there is no significant effect of the CCyB on the mortgage LTV distribution, which is in contrast to the significant effects in this paper. My analysis differs in three respects from these studies, as follows: (i) the sample size and period, (ii) the measurement of the outcome variable and (iii) the definition of the CCyB treatment group.

Regarding the work by Auer and Ongena (2019), the differences in the sample size and period do not drive the different results. If I run my main regression with the 20 banks of the Auer-Ongena sample and/or restrict the sample period to be the same period as theirs (2012Q3-2013Q4) (see Table A.10), I find similar (non-)results to those reported in Table 3. Instead, the different effects on commercial loans are likely driven by how this outcome variable is measured; even if I use the Auer-Ongena treatment definition, I still find insignificant effects (see Table A.10). My paper relies on the change in the stock of commercial loans reported in the balance sheet. Auer-Ongena measure the flow of new lending based on loan-level data. While preferable in principle, the latter is potentially problematic in the Swiss context due to measurement issues.^15^

Moreover, there are differences in the treatment definition; Auer-Ongena use the ratio of residential mortgage RWA over domestic assets to determine the CCyB exposure, without taking into account the bank’s excess capital. This leads to the differences illustrated in Table 2; i.e., the well capitalised, high mortgage risk bank (second row) would be in my control but their treatment group, whereas the tightly capitalised, low mortgage risk bank (third row) would be in my treatment but their control group. While there is a positive rank correlation of 0.55 between my CCyB treatment intensity variable and theirs, using their definition would “re-classify” 13 banks in my sample from treatment to control or vice-versa. Given these differences, it is no surprise that the effects of the RRWA/asset ratio are not similar to the CCyB treatment effects, i.e., most of the effects are insignificant. My interpretation is that the banks with higher residential RWA/asset ratios did not behave differently compared to banks with lower ratios, but this ratio is not a good proxy for being affected by the CCyB activation.

Comparing my results to Basten (2020), differences in the sample size and period do not drive differences in statistical significance; i.e., if I run my main regression with the 7 banks that are in both samples and/or restrict the sample period to be the same as his (2012Q3-2013Q3), the estimated effects on the LTV distribution and mortgage growth rates remain statistically significant (see Table A.11). Another possible driver is the difference in measurement of CCyB exposure. Using his approach, though, results in a similar treatment group.^16^ Hence, using his treatment definition does not materially affect the results in my sample. Instead, the difference in statistical significance could be driven by the precision with which the LTV ratio is measured: I observe the LTV distribution once the mortgage contract has been signed, while Basten (2020) observes LTVs at the time of the borrower request on an online platform.

## Conclusion

In this paper, I have shown that both macroprudential measures introduced in Switzerland in 2012/13 contributed to lean against a further accumulation of mortgage risks. They significantly reduced the LTV risks of residential mortgages by affecting different parts of the LTV distribution. The banks more affected by the LTV cap reduced the share of new mortgages with LTV ratios over 90% (and hence complied with the LTV cap). The banks more affected by the CCyB reduced their share of mortgages with LTV ratios over 80% (that receive a higher risk-weight according to the standardised approach of capital regulation). Moreover, some of the banks affected by the CCyB reduced their mortgage RWAs by reducing their mortgage growth rates. The economic magnitude of the estimated effects is substantial.

In contrast to other studies, I do not find that banks with high treatment intensities increased observable risk-taking: they did not issue more high LTI mortgages, more EtP mortgages or more non-mortgage loans to firms. However, in the banking system as a whole, the reduction in LTV risks was accompanied by an increase in LTI risks. My cross-sectional evidence suggests that the increase in LTI risk was not caused by the macroprudential policies, but it was also not prevented by them.

While the macroprudential measures contributed to financial stability, mortgage borrowers and banks were restricted in their choice set. Their reaction to the LTV cap was rather homogenous, but did affect only a small part of the LTV distribution of new mortgages. While the LTV cap resulted in an immediate risk reduction in Switzerland, evidence on spill-over effects from other countries suggests that such caps must be carefully designed and intensely monitored.

The CCyB did not directly restrict banks in issuing mortgages with certain characteristics, but set incentives to reduce risk-weighted assets. Thus, banks’ reaction to the CCyB activation was more heterogenous: banks compressed different parts of their LTV distribution and only a few banks reduced their mortgage growth rates. Since many banks in Switzerland are well capitalised, the CCyB affected only a few banks, though. Hence, my CCyB estimates have the same direction, but a smaller magnitude compared to the simulation for the UK banking system by Noss and Toffano (2016).

The effect of the CCyB on the overall resilience of the banking system lies beyond the scope this paper. Increased capital buffers enhance the survival probability of banks during banking crises (Berger and Bouwman, 2013). Higher capital is also associated with greater lending because better capitalised banks face lower funding costs (Gambacorta and Shin, 2018). The CCyB, though, is no panacea: it can contribute to the resilience of the banking system, but does not prevent credit booms and asset prices bust (Allen and Gu, 2018). Another rationale for a countercyclical capital buffer – not analysed in this paper - is that eliminates market failure caused by asymmetric information between banks and entrepreneurs (Jokivuolle et al., 2013).

In March 2020, the CCyB was deactivated in spite of the continuing vulnerabilities in the mortgage market. This decision was made against the backdrop of the coronavirus crisis in order to broaden the scope of banks to lend to companies in these exceptional circumstances (Zurbrügg, 2021). Since then mortgage lending has increased. It remains to be analysed how much of this increase is related to supply and demand effects. On the one hand, banks could issue mortgages at lower capital costs after the CCyB was released. On the other hand, preferences for housing might have risen in the pandemic.

## Supplementary Information

ESM 1 (DOCX 0.99 kb)

## Footnotes

1 Aside from Switzerland, the CCyB has been implemented in a number of other countries, i.e., Sweden and Norway in 2015, Hong Kong and the UK in 2016, the Czech Republic, Iceland and Slovakia in 2017, Ireland in 2018, France and Germany in 2019, and Luxembourg in 2020.

2 Blatter and Fuster (2021), Beutler et al. (2020), Auer et al. (2017), Dietrich (2016), and Vujanovic (2016) provide a further description of the Swiss banking and housing sector.

3 Swiss covered bonds serve refinancing purposes, but no risk transfer takes place.

4 Under the standardised approach, a mortgage tranche with LTV ratio <66% has a risk-weight of 35%, one with LTV ratio between 66%- 80% has a risk-weight of 75% and one with LTV ratio >80% has a risk-weight of 100%. For illustration purposes, let us consider a loan close to 900,000 CHF for a house with value of 1,000,000 CHF. For a loan of 899,900, the residential RWA is 433,332 (the average risk-weight is 48%), while for a loan of 900,001, the residential RWA is 900,001 (the average risk-weight is 100%).

5 Risk-sensitive capital requirements may contribute to cyclicality of credit supply; higher capital requirements in booms may contain excessive lending. Jokivuolle et al. (2014) provide another rationale for countercyclical risk-sensitive capital requirements: they may reduce market failure (overinvestment in risky projects) in the presence of adverse selection.

6 An earlier version of the amortisation requirement was implemented in the first revision of the self-regulation, where mortgage borrowers were asked to amortise their loan to an LTV of 2/3 within the next 20 years. However, this version was not binding, because the amortisation amount and schedule were looser than the banks’ practice.

7 Besides the effects on LTVs, there could be an effect on LTIs that are a key determinant for banks using the IRB approach. However, only 3 banks in my sample apply the IRB approach. Hence, I am less likely to observe changes in the LTI distribution.

8 There is also no evidence that foreign policies affect domestic lending (Auer and al., 2017).

9 Banks report the market values of houses according to a standardised definition. Therefore, it should not depend on how conservative a bank is in the valuation process of the house. The banks did not change their market value reporting behaviour during the sample period. In particular, the distribution of the market value did not change for my treatment groups after treatment; i.e., if I run my main regression on selected descriptive statistics of the market value distribution, I do not find any statistically significant effects

10 In Switzerland, one third of gross income corresponds to 38% percent of net income (reported in the survey), while the mortgage cost are assumed 7% (5% interest rate payment + 1% amortisation + 1% maintenance) resp. 5% (3% interest rate payment + 1% amortisation + 1% maintenance) of the mortgage loan, resulting in an LTI threshold of 38/7= 5.4 resp. 38/5= 7.6.

11 If I use end-2011 values to define treatment exposure, the results remain qualitatively similar but are less precisely estimated due to the measurement error introduced.

12 I find similar effects if I consider different LTV buckets: for over 80% the coefficient is −1.88; for 70%–80% it is 1.95.

13 Similarly, not every bank adjusted its LTV distribution in the same way as a reaction to the CCyB activation.

14 Further robustness checks – such as including other time-varying covariates, estimating short- medium and long term effects and placebo treatment effects - are in the working paper version of this paper (Behncke 2020).

15 In particular, the available dataset does not reliably distinguish between a true new loan and a change in conditions of an existing loan (see also Schelling and Towbin, 2020).

16 Basten assumes that banks with below median capital cushions and above median mortgage/asset ratio are affected by the CCyB. Although his definition is different from mine, the treatment groups happen to be similar. Three of my four treated banks would also be in his treatment group. This overlap in treatment groups depends on a specific feature in his sample, i.e., banks have smaller capital cushions. Because their median value corresponds to the 20th percentile of my sample, it is informative for identifying tightly capitalised banks.

## References

[CR1] Acharya V, Bergant K, Crosignani M, Eisert T, McCann F (2021). The anatomy of the transmission of macroprudential policies.

[CR2] Aiyar, Shekhar, Charles W. Calomiris, and Tomasz Wieladek. (2014). “Does Macro-Prudential Regulation Leak? Evidence from a UK Policy Experiment,” *Journal of Money,* Credit and Banking, 46(s1), 181–214

[CR3] Akinci O, Olmstead-Rumsey J (2018). How effective are macroprudential policies? An empirical investigation. J Financ Intermed.

[CR4] Allen J, Grieder T, Peterson B, Roberts T (2020) The impact of macroprudential housing finance tools in Canada. J Financ Intermed 42

[CR5] Allen F, Gu X (2018). The interplay between regulations and financial stability. Journal Financial Service Research.

[CR6] Arnold B, Borio C, Luciellis FM (2012). Systemic risk, macroprudential policy frameworks, monitoring financial systems and the evolution of capital adequacy. J Bank Financ.

[CR7] Auer, Raphael, and Steven Ongena. (2019). “The countercyclical capital buffer and the composition of bank lending.” *CEPR Discussion Paper No. 13942*

[CR8] Auer S, Ganarin M, Towbin P (2017). International banking and cross-border effects of regulation: lessons from Switzerland. Int J Cent Bank.

[CR9] Basel Committee on Banking Supervision (BCBS) (2017). “*Implementation. Range of practices in implementing the countercyclical buffer policy*”

[CR10] Basel Committee on Banking Supervision (BCBS) (2018). “Towards a sectoral application of the countercyclical capital buffer: A literature review“, *Working Papers 32*

[CR11] Basten C (2020). Higher bank capital requirements and mortgage pricing: evidence from the counter-cyclical capital buffer. Review of Finance.

[CR12] Basten C, Koch C (2015). Higher bank capital requirements and mortgage pricing: evidence from the countercyclical capital buffer (CCyB). BIS working papers 511.

[CR13] Behncke S (2020). “Effects of macroprudential policies on Bank lending and credit risks”, *SNB working paper*.

[CR14] Behncke S (2012). How do shocks to non-cognitive skills affect test scores?. Annals of Economics and Statistics.

[CR15] Berger A, Bouwman C (2013). How does capital affect bank performance during financial crises?. J Financ Econ.

[CR16] Beutler T, Bichsel R, Bruhin A, Danton J (2020) The impact of interest rate risk on Bank lending. J Bank Financ 115(C)

[CR17] Blatter, Marc and Andreas Fuster (2021). “Scale Effects on Efficiency and Profitability in the Swiss Banking Sector”, *SNB working paper*10.1186/s41937-022-00091-7 PMC9080912 35573144

[CR18] Buch C, Goldberg L (2017). Cross-border Prudential policy spillovers: how much? How important? Evidence from the international banking research network. Int J Cent Bank.

[CR19] Cameron AC, Gelbach JB, Miller DL (2008). Bootstrap-based improvements for inference with clustered errors. Rev Econ Stat.

[CR20] Cerutti E, Claessens S, Laeven L (2017). The use and effectiveness of macroprudential policies: new evidence. J Financ Stab.

[CR21] Claessens, Stijn, Giovanni Dell’Ariccia, Deniz Igan, and Luc Laeven. (2010). “Lessons and Policy Implications from the Global Financial Crisis,” *IMF working paper* 10/44

[CR22] Claessens S (2015). An overview of macroprudential policy tools. Annual Review of Financial Economics.

[CR23] Dietrich A (2016). What drives the gross margins of mortgage loans? Evidence from Switzerland. Journal of Financial Service Research.

[CR24] Drehmann M, Gambacorta L (2012). The effects of countercyclical capital buffers on bank lending. Appl Econ Lett.

[CR25] Gambacorta L, Mistrulli PE (2004). Does bank capital affect lending behavior?. J Financ Intermed.

[CR26] Gambacorta L, Shin HS (2018). Why bank capital matters for monetary policy. J Financ Intermed.

[CR27] Igan D, Kang H (2011). “Do loan-to-value and debt-to-income limits work? Evidence from Korea”, no 11/297.

[CR28] Jimenez G, Ongena S, Peydro J-L, Saurina J (2017). Macroprudential policy, countercyclical Bank capital buffers and credit supply: evidence from the Spanish dynamic provisioning experiments. J Polit Econ.

[CR29] Jokivuolle E, Kiema I, Vesala T (2014). Why do we need countercyclical capital requirements?. Journal Financial Service Research.

[CR30] Juelsrud R, Wold E (2020) Risk-weighted capital requirements and portfolio rebalancing. J Financ Intermed 41(C)

[CR31] Kim S, Plosser M, Santos J (2018). Macroprudential policy and the revolving door of risk: lessons from leveraged lending guidance. J Financ Intermed.

[CR32] Kinghan, Christina, Yvonne McCarthy, and Conor O’Toole. (2019)” How do macroprudential loan-to-value restrictions impact first time home buyers? A quasi-experimental approach.” *Journal of Banking and Finance*, in press

[CR33] Lim CH, Costa A, Columba F, Kongsamut P, Otani A, Saiyid M, Wezel T, Wu X (2011). Macroprudential policy: what instruments and how to use them? Lessons from country experiences. *IMF working papers* 11/238.

[CR34] McCann F, O’Toole C (2019). Cross-border macroprudential policy spillovers and Bank risk-taking. Int J Cent Bank.

[CR35] Morgan P, Regis P, Salike N (2019). LTV policy as a macroprudential tool and its effects on residential mortgage loans. J Financ Intermed.

[CR36] Noss J, Toffano P (2016). Estimating the impact of changes in aggregate bank capital requirement on lending and growth during an upswing. J Bank Financ.

[CR37] Schelling T, Towbin P (2020). Negative interest rates, deposit funding and bank lending.

[CR38] Seiler Y (2013). Nutzung von Vorsorgegeldern zur Finanzierung von selbstgenutztem Wohneigentum.

[CR39] SNB. (2014). Factsheet: Implementing the countercyclical capital buffer in Switzerland: concretising the Swiss National Bank’s role

[CR40] Tzur-Ilan, Nitzan. (2020) “The real consequences of LTV limits on housing choices.” working paper

[CR41] Uluc A, Wieladek T (2018). Capital requirements, monetary policy and risk shifting in the mortgage market. J Financ Intermed.

[CR42] Vujanovic P (2016). Policies to tame the housing cycle in Switzerland.

[CR43] Wong, Eric, Fong, Tom, Li, Ka Fai, and Choi, Henry. (2011), “Loan-to-Value Ratio as a Macro-Prudential Tool - Hong Kong's Experience and Cross-Country Evidence,” No 1101, Working Papers*,* Hong Kong Monetary Authority

[CR44] Zurbrügg F (2021) Mortgage and real estate markets: current developments pose risks to financial stability. SNB

