# Supplementary material for: Effects of Macroprudential Policies on Bank Lending and Credit Risks
Source: J Financ Serv Res. 2022 Mar 24;63(2):175–99. doi: 10.1007/s10693-022-00378-z (PMC8943354; doi:10.1007/s10693-022-00378-z)
Supplement: Supplementary file 1 — (DOCX 0.99 kb) [file 10693_2022_378_MOESM1_ESM.docx]

# Appendix


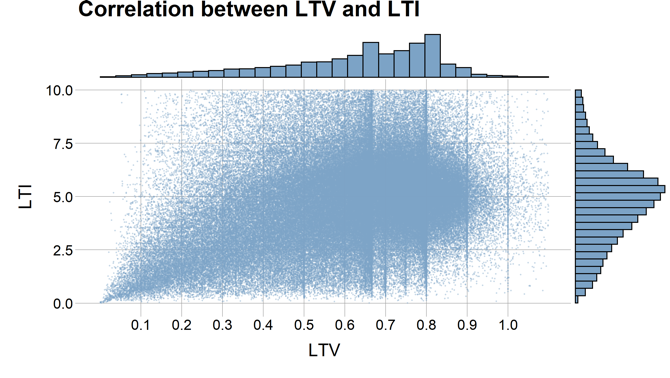

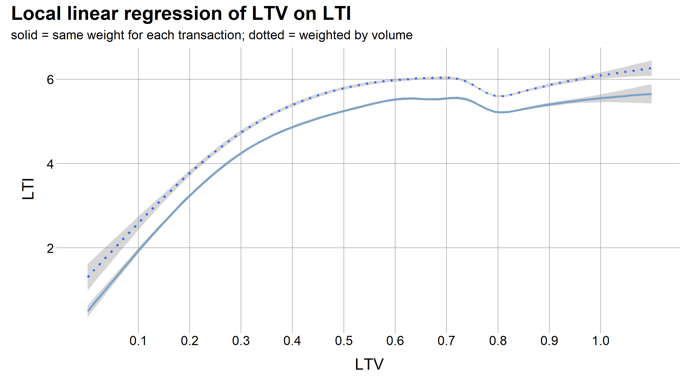


**Figure A.1** Evidence on the correlation between LTV and LTI from a loan-level survey. The correlation between the LTV and the LTI ratio is measured based on a loan-level survey (HYPO_B). It covers the sample period 2017-2020. The definition of LTV, LTI of new mortgages to private households are identical to the definition in the mortgage survey used in this paper. The sample consists of the same 25 banks.


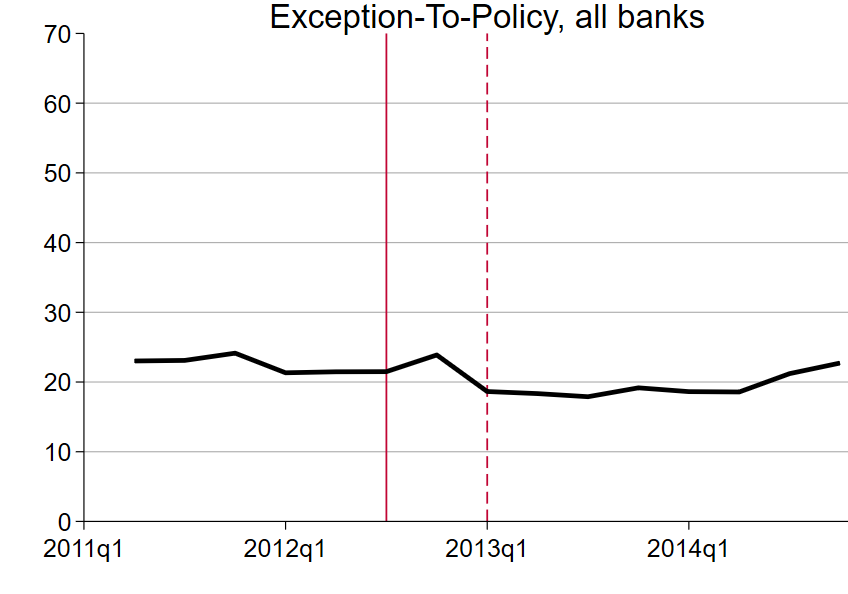

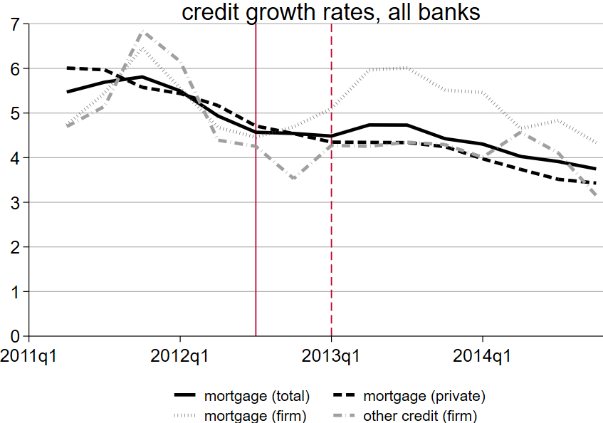


**Figure A.2** share of new EtP mortgages (left) and credit growth rates (right) (2011Q2-2014Q4). The vertical lines indicate the implementation of the LTV cap (2012Q3) and the activation of the CCyB (2013Q1).

**Table A.1:** Descriptive statistics

|  | **N** | **Mean** | **Std. Dev.** | **p10** | **p50** | **p90** |
| --- | --- | --- | --- | --- | --- | --- |
| **outcome variables** |  |  |  |  |  |  |
| share of new mortgages with LTV>90% | 600 | 2.55 | 3.09 | 0 | 1 | 7 |
| LTV between 80%-90% | 600 | 14.52 | 6.63 | 5 | 14 | 23 |
| LTV between 66%-80% | 600 | 38.30 | 6.80 | 30 | 38 | 47 |
| LTI very high (>7.6) | 575 | 14.88 | 9.42 | 5 | 13 | 28 |
| LTI high (between 5.4 and 7.6) | 575 | 24.59 | 7.26 | 14 | 25 | 33 |
| EtP share | 600 | 21.25 | 13.58 | 4 | 21 | 38 |
| mortgage growth rate, total | 850 | 4.61 | 2.41 | 1.63 | 4.58 | 7.62 |
| mortgage growth rate, households | 850 | 4.61 | 2.75 | 1.63 | 4.25 | 7.89 |
| mortgage growth rate, firms | 850 | 4.81 | 5.08 | -0.78 | 4.40 | 10.80 |
| other credit growth rate, firms | 850 | 4.09 | 5.92 | -2.81 | 3.86 | 11.34 |
| **treatment variables** |  |  |  |  |  |  |
| LTV_exp_ (before standardisation) | 25 | 4.33 | 3.18 | 0 | 4.23 | 8.69 |
| CCyB_exp_ (before standardisation) | 25 | 0.37 | 0.92 | 0.05 | 0.12 | 0.62 |

**Table A.2** Effect heterogeneity for the bank with the highest CCyB treatment intensity. All regressions control for bank and quarter time fixed effects. Standard errors are determined by a wild cluster bootstrap with ***,**,* denoting significance at the 1%, 5% and 10% levels, respectively. *CCyB_bank1_* resp. *CCyB_bank2-4_* are dummies for the bank with the highest treatment intensity resp. for the other three banks in the *CCyB_dum_* treatment group. *T_2012Q3_* resp. *T_2013Q1_* are time dummies equal to 1 from 2012Q3 resp. 2013Q1 onward. EtP denotes Exception-to-Policy mortgages.

|  | **Mortgage share with LTV** | | | | **…with LTI** | | **with** | **Mortgage growth** | | | **credit** |
| --- | --- | --- | --- | --- | --- | --- | --- | --- | --- | --- | --- |
|  | **>90%** | **80-90%** | **66-80%** | **<66%** | **>7.6** | **5.4-7.6** | **EtP** | **total** | **private** | **firms** | **firms** |
|  |  |  |  |  |  |  |  |  |  |  |  |
| **LTV_dum_*T_2012Q3_** | -4.37*** | -0.07 | 2.41 | 2.04 | -0.88 | -1.30 | -6.91 | -0.84 | -0.66 | -0.83 | 0.18 |
|  | (0.73) | (1.92) | (1.90) | (1.35) | (2.25) | (1.71) | (4.18) | (0.72) | (0.85) | (1.39) | (1.52) |
| **CCyB_bank2-4_* T_2013Q1_** | -0.45 | -2.22* | 9.24*** | -6.56*** | -4.73 | 1.81 | -3.45 | -2.39*** | -3.00** | -1.47* | 2.06 |
|  | (0.72) | (1.15) | (1.49) | (0.96) | (3.36) | (1.54) | (4.19) | (0.54) | (0.76) | (0.83) | (1.59) |
| **CCyB_bank1_* T_2013Q1_** | 0.06 | -5.28*** | 1.48 | 6.70*** | 4.91 | -7.81*** | 2.17 | 1.44*** | 1.95*** | 0.75 | -1.73 |
|  | (0.62) | (0.65) | (1.34) | (0.72) | (3.19) | (1.40) | (3.03) | (0.31) | (0.64) | (0.55) | (1.69) |
|  |  |  |  |  |  |  |  |  |  |  |  |
| Observations | 600 | 600 | 600 | 600 | 575 | 575 | 600 | 850 | 850 | 850 | 850 |
| R-squared | 0.55 | 0.58 | 0.44 | 0.67 | 0.61 | 0.54 | 0.60 | 0.60 | 0.62 | 0.50 | 0.38 |
| Mean (dep. var.) | 2.55 | 14.52 | 38.30 | 44.63 | 14.88 | 24.59 | 21.25 | 4.61 | 4.61 | 4.81 | 4.09 |
| Std (dep. var.) | 3.09 | 6.63 | 6.80 | 7.73 | 9.42 | 7.26 | 13.58 | 2.41 | 2.75 | 5.08 | 5.92 |
|  |  |  |  |  |  |  |  |  |  |  |  |

Standard errors are determined by a wild cluster bootstrap with ***,**,* denoting significance at the 1%, 5% and 10% levels, respectively.

**Table A.3** Rolling group test: banks with the highest LTV treatment intensity. All regressions control for bank and quarter time fixed effects. *LTV_ibanks_* measures whether a bank belongs to the *i* banks with the highest LTV_exp_ (LTV treatment intensity). *CCyB_4banks_* is equivalent to the *CCyB_dum_* treatment group. *T_2012Q3_* resp. *T_2013Q1_* are time dummies equal to 1 from 2012Q3 resp. 2013Q1 onward. EtP denotes Exception-to-Policy mortgages. For the LTV treatment definition, I select the three/six/nine/twelve/fifteen banks with the highest treatment definition. Results are stable across specifications. They indicate that the share of new mortgages with LTV ratios of more than 90% is around 4 percentage points smaller in the treatment compared to the control group after the implementation of the LTV cap.

|  | **Mortgage share with LTV** | | | | **… with LTI** | | **with** | **Mortgage growth** | | | **credit** |
| --- | --- | --- | --- | --- | --- | --- | --- | --- | --- | --- | --- |
|  | **>90%** | **80-90%** | **66-80%** | **<66%** | **>7.6** | **5.4-7.6** | **EtP** | **total** | **private** | **firms** | **firms** |
|  |  |  |  |  |  |  |  |  |  |  |  |
| **3 banks with highest LTV treatment intensity** | | | | | | | | | | | |
| **LTV3banks*T_2012Q3_** | -4.23*** | -1.77 | 3.75** | 2.25 | -1.89 | 1.48 | -7.16 | -1.14 | -1.87 | -0.39 | 2.91* |
|  | (0.63) | (2.58) | (1.49) | (1.55) | (2.78) | (1.72) | (5.92) | (1.42) | (1.19) | (2.27) | (1.44) |
| **CCyB4banks*T_2013Q1_** | -0.97 | -3.36* | 9.01*** | -4.67** | -3.48 | -0.57 | -1.73 | -2.11*** | -2.44*** | -1.47 | 1.28 |
|  | (1.05) | (1.76) | (1.21) | (1.95) | (2.56) | (2.28) | (4.79) | (0.60) | (0.69) | (0.86) | (0.95) |
| **6 banks with highest LTV treatment intensity** | | | | | | | | | | | |
| **LTV6banks*T_2012Q3_** | -3.73*** | -2.24 | 2.30 | 3.67** | -3.49 | 0.07 | -7.96 | -1.25 | -1.83* | -1.38 | -1.07 |
|  | (0.85) | (1.91) | (1.82) | (1.53) | (2.70) | (1.85) | (5.44) | (0.80) | (0.94) | (1.50) | (2.06) |
| **CCyB4banks*T_2013Q1_** | -1.41 | -3.54* | 9.40*** | -4.45** | -3.69 | -0.42 | -3.56 | -2.24*** | -2.68*** | -1.51* | 1.69 |
|  | (1.03) | (1.75) | (1.34) | (2.07) | (2.39) | (2.30) | (4.01) | (0.53) | (0.65) | (0.79) | (1.66) |
| **9 banks with highest LTV treatment intensity** | | | | | | | | | | | |
| **LTV9banks*T_2012Q3_** | -4.58*** | -0.91 | 3.53** | 1.96 | -1.48 | -0.18 | -7.43 | -1.01 | -0.94 | -0.82 | -0.19 |
|  | (0.70) | (1.79) | (1.70) | (1.52) | (2.37) | (1.72) | (4.37) | (0.81) | (0.91) | (1.36) | (1.65) |
| **CCyB4banks*T_2013Q1_** | -0.90 | -3.45* | 9.00*** | -4.65** | -3.53 | -0.40 | -2.74 | -2.12*** | -2.56*** | -1.40* | 1.70 |
|  | (0.92) | (1.75) | (1.19) | (2.05) | (2.73) | (2.36) | (3.59) | (0.47) | (0.68) | (0.80) | (1.53) |
| **Baseline dummy specification: 12 banks with highest treatment intensity** | | | | | | | | | | | |
| **LTV12banks*T_2012Q3_** | -4.37*** | -0.27 | 2.35 | 2.28 | -0.69 | -1.61 | -6.70 | -0.79 | -0.59 | -0.81 | 0.12 |
|  | (0.72) | (1.90) | (1.88) | (1.35) | (2.21) | (1.75) | (4.19) | (0.72) | (0.84) | (1.39) | (1.52) |
| **CCyB4banks*T_2013Q1_** | -0.44 | -3.50** | 8.88*** | -4.95*** | -3.54 | -0.08 | -2.09 | -2.04*** | -2.53*** | -1.29 | 1.64 |
|  | (0.62) | (1.59) | (1.29) | (1.72) | (2.86) | (2.17) | (3.87) | (0.59) | (0.80) | (0.79) | (1.54) |
| **15 banks with highest treatment intensity** | | | | | | | | | | | |
| **LTV15banks*T_2012Q3_** | -3.54*** | 0.64 | 0.83 | 2.07 | -1.85 | -1.37 | -1.90 | -0.46 | -1.21 | 1.68 | 1.82 |
|  | (0.83) | (2.22) | (2.21) | (1.38) | (2.32) | (2.02) | (4.35) | (0.63) | (0.74) | (1.41) | (1.34) |
| **CCyB4banks*T_2013Q1_** | -0.99 | -3.64** | 9.32*** | -4.69** | -3.49 | -0.27 | -3.39 | -2.19*** | -2.50*** | -1.78** | 1.39 |
|  | (0.73) | (1.60) | (1.44) | (1.71) | (2.77) | (2.18) | (3.41) | (0.57) | (0.77) | (0.81) | (1.43) |
|  |  |  |  |  |  |  |  |  |  |  |  |
| Observations | 600 | 600 | 600 | 600 | 575 | 575 | 600 | 850 | 850 | 850 | 850 |
| Mean (dep. var.) | 2.55 | 14.52 | 38.30 | 44.63 | 14.88 | 24.59 | 21.25 | 4.61 | 4.61 | 4.81 | 4.09 |
| Std (dep. var.) | 3.09 | 6.63 | 6.80 | 7.73 | 9.42 | 7.26 | 13.58 | 2.41 | 2.75 | 5.08 | 5.92 |
|  |  |  |  |  |  |  |  |  |  |  |  |

Standard errors are determined by a wild cluster bootstrap with ***,**,* denoting significance at the 1%, 5% and 10% levels, respectively.

**Table A.4** Rolling group test: banks with the highest CCyB treatment intensity. All regressions control for bank and quarter time fixed effects. *CCyB_ibanks_* measures whether a bank belongs to the *i* banks with the highest CCyB_exp_ (CCyB treatment intensity). *LTV_12banks_* is equivalent to the *LTV_dum_* treatment group. *T_2012Q3_* resp. *T_2013Q1_* are time dummies equal to 1 from 2012Q3 resp. 2013Q1 onward. EtP denotes Exception-to-Policy mortgages. Because there are fewer banks with a high CCyB treatment intensity compared to the LTV treatment intensity, I select the three/four/five/six banks with the highest treatment intensity. Results are qualitatively similar across specifications. (One exception is the five-bank specification due to effect heterogeneity of the fifths bank). Banks in the CCyB treatment group reduce the share of the 80%-90% LTV bucket and increase the share of the 66%-80% LTV bucket. Banks reduce their mortgage growth rates by around 2 percentage points after the CCyB activation**.**

|  | **Mortgage share with LTV** | | | | **…with LTI** | | **with** | **Mortgage growth** | | | **credit** |
| --- | --- | --- | --- | --- | --- | --- | --- | --- | --- | --- | --- |
|  | **>90%** | **80-90%** | **66-80%** | **<66%** | **>7.6** | **5.4-7.6** | **EtP** | **total** | **private** | **firms** | **firms** |
|  |  |  |  |  |  |  |  |  |  |  |  |
| **3 banks with highest CCyB treatment intensity** | | | | | | | | | | | |
| **LTV12banks*T_2012Q3_** | -4.44*** | -0.51 | 3.15 | 1.80 | -1.21 | -1.52 | -6.67 | -0.96 | -0.83 | -0.91 | 0.38 |
|  | (0.70) | (1.90) | (2.09) | (1.44) | (2.27) | (1.75) | (4.04) | (0.74) | (0.90) | (1.38) | (1.50) |
| **CCyB3banks*T_2013Q1_** | 0.04 | -3.66* | 7.15*** | -3.53* | -0.01 | -1.34 | -4.36 | -1.74*** | -1.78** | -1.19 | -0.08 |
|  | (0.53) | (1.86) | (1.08) | (1.94) | (1.35) | (2.48) | (3.59) | (0.62) | (0.76) | (0.82) | (0.88) |
| **Baseline specification: 4 banks with highest CCyB treatment intensity** | | | | | | | | | | | |
| **LTV12banks*T_2012Q3_** | -4.37*** | -0.27 | 2.35 | 2.28 | -0.69 | -1.61 | -6.70 | -0.79 | -0.59 | -0.81 | 0.12 |
|  | (0.72) | (1.90) | (1.88) | (1.35) | (2.21) | (1.75) | (4.19) | (0.72) | (0.84) | (1.39) | (1.52) |
| **CCyB4banks*T_2013Q1_** | -0.44 | -3.50** | 8.88*** | -4.95*** | -3.54 | -0.08 | -2.09 | -2.04*** | -2.53*** | -1.29 | 1.64 |
|  | (0.62) | (1.59) | (1.29) | (1.72) | (2.86) | (2.17) | (3.87) | (0.59) | (0.80) | (0.79) | (1.54) |
| **5 banks with highest CCyB treatment intensity** | | | | | | | | | | | |
| **LTV12banks*T_2012Q3_** | -4.47*** | -0.39 | 2.28 | 2.58* | -0.64 | -1.56 | -6.92 | -0.74 | -0.52 | -0.79 | -0.10 |
|  | (0.72) | (1.90) | (1.93) | (1.39) | (2.24) | (1.78) | (4.36) | (0.77) | (0.88) | (1.43) | (1.57) |
| **CCyB5banks*T_2013Q1_** | 0.13 | -1.83 | 6.33** | -4.62*** | -2.58 | -0.26 | -0.43 | -1.58** | -2.00** | -0.93 | 2.05 |
|  | (0.69) | (1.95) | (2.45) | (1.55) | (2.59) | (1.86) | (3.97) | (0.73) | (0.89) | (0.89) | (1.45) |
| **6 banks with highest CCyB treatment intensity** | | | | | | | | | | | |
| **LTV12banks*T_2012Q3_** | -4.42*** | 0.20 | 1.75 | 2.47 | -0.49 | -1.51 | -7.54 | -0.68 | -0.39 | -0.83 | -0.17 |
|  | (0.76) | (1.79) | (1.90) | (1.45) | (2.31) | (1.83) | (4.52) | (0.83) | (0.92) | (1.48) | (1.64) |
| **CCyB6banks*T_2013Q1_** | -0.04 | -3.30* | 6.49*** | -3.15 | -2.43 | -0.35 | 1.71 | -1.41* | -1.92** | -0.59 | 1.77 |
|  | (0.67) | (1.86) | (2.10) | (1.84) | (2.38) | (1.70) | (4.19) | (0.77) | (0.87) | (0.99) | (1.46) |
|  |  |  |  |  |  |  |  |  |  |  |  |
| Observations | 600 | 600 | 600 | 600 | 575 | 575 | 600 | 850 | 850 | 850 | 850 |
| Mean (dep. var.) | 2.55 | 14.52 | 38.30 | 44.63 | 14.88 | 24.59 | 21.25 | 4.61 | 4.61 | 4.81 | 4.09 |
| Std (dep. var.) | 3.09 | 6.63 | 6.80 | 7.73 | 9.42 | 7.26 | 13.58 | 2.41 | 2.75 | 5.08 | 5.92 |
|  |  |  |  |  |  |  |  |  |  |  |  |

Standard errors are determined by a wild cluster bootstrap with ***,**,* denoting significance at the 1%, 5% and 10% levels, respectively.


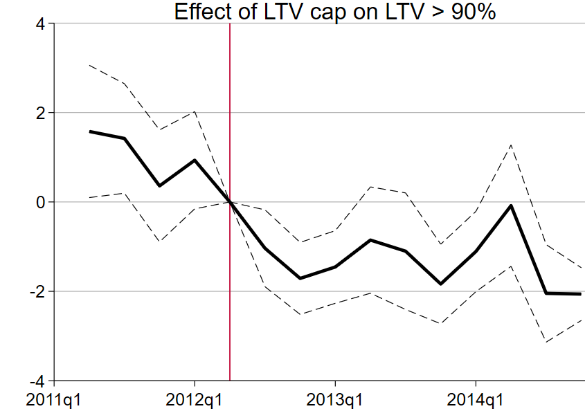

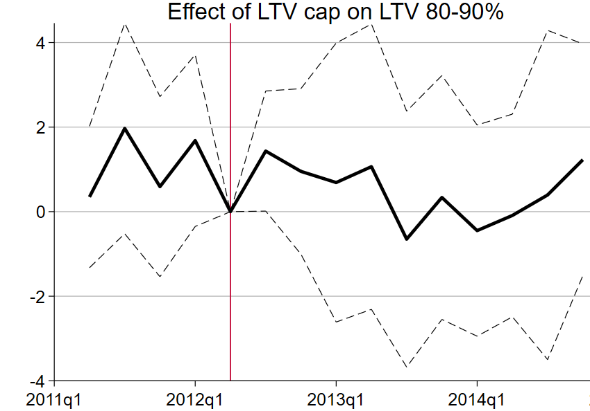


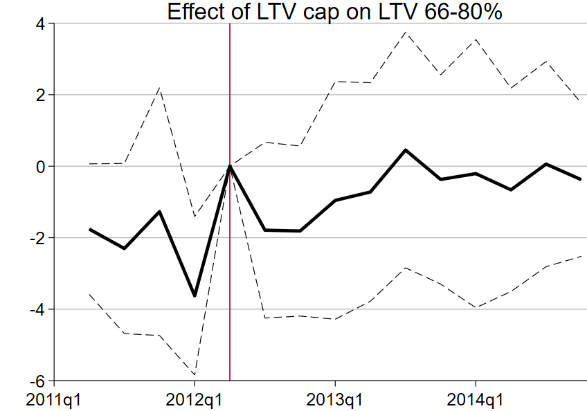

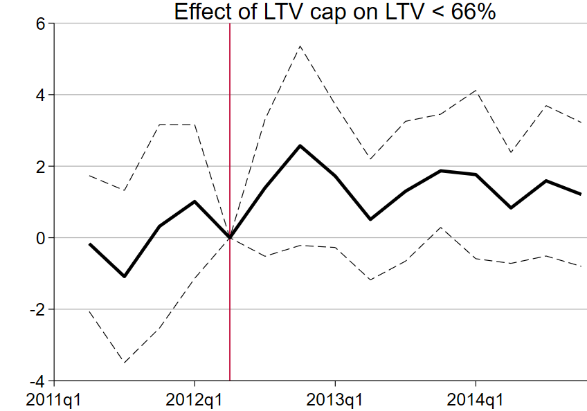


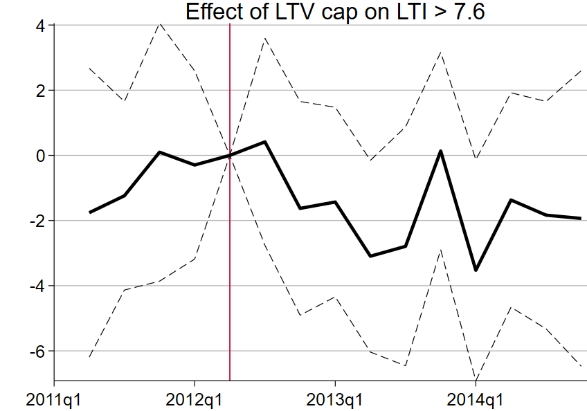

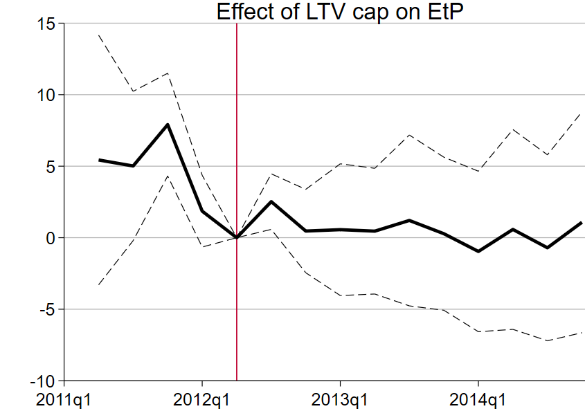


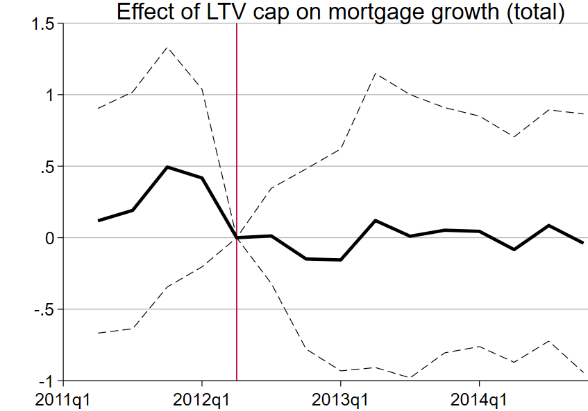

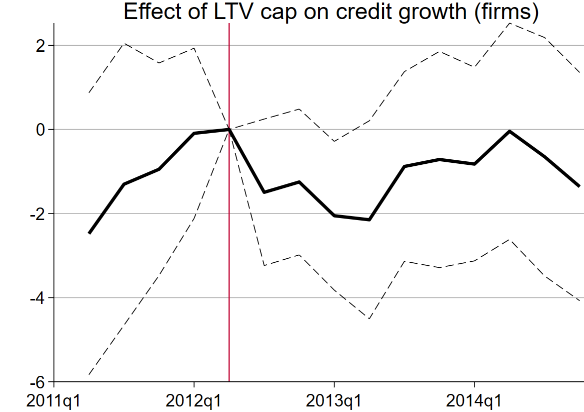


**Figure A2** Dynamic difference-in-differences, by LTV treatment intensity. See Note below Figure 2.


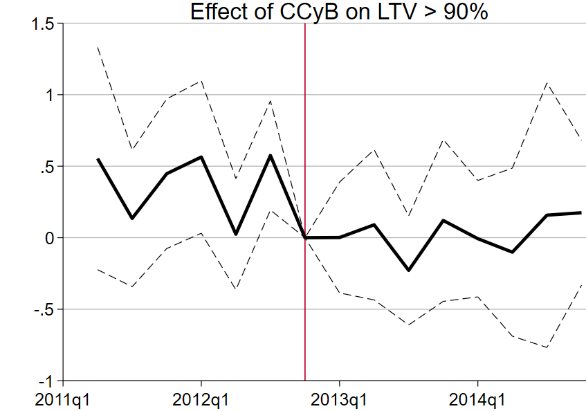

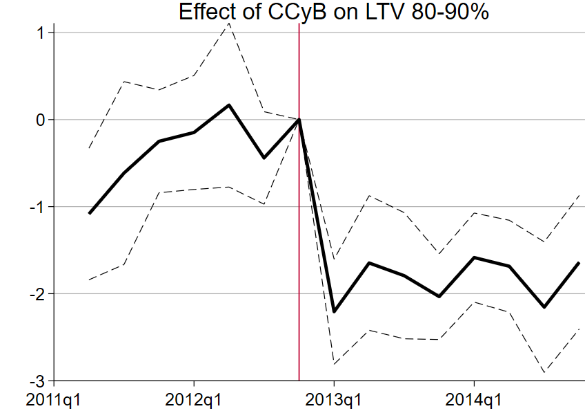


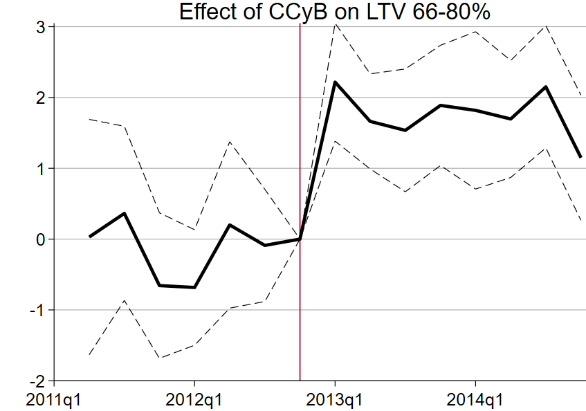

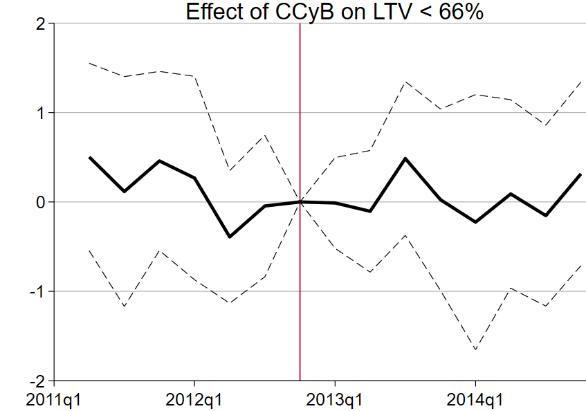


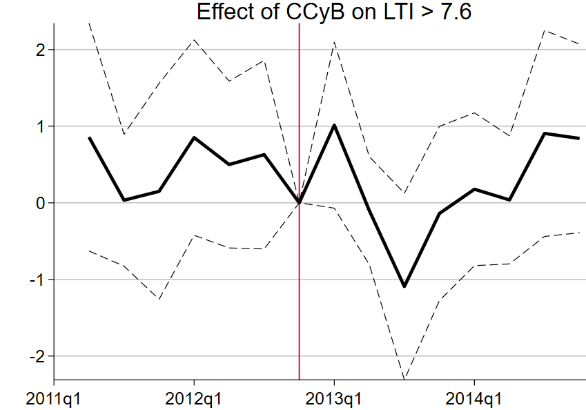

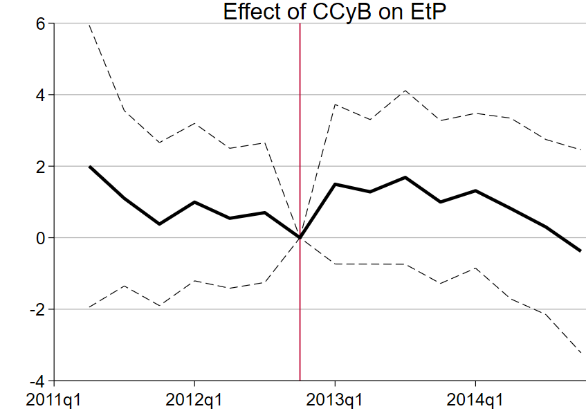


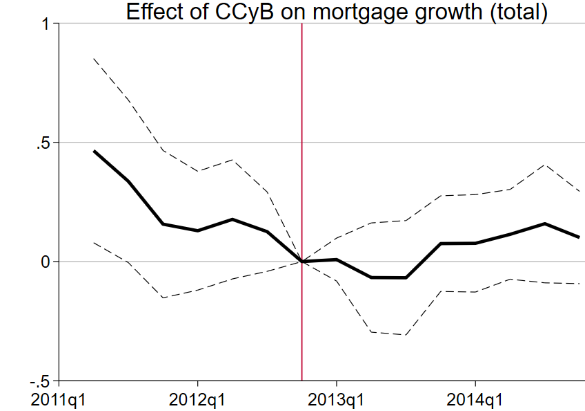

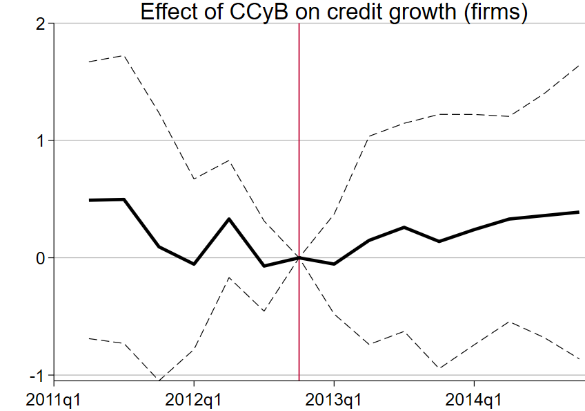


**Figure A3** Dynamic difference-in-differences, by CCyB treatment intensity. See Note below Figure 2.

**Table A.5** Anticipation of LTV cap in 2012Q2. All regressions control for bank and quarter time fixed effects. *LTV_exp_* and *CCyB*_exp_ measure the standardised treatment intensities of the LTV cap and the CCyB activation. *T_2012Q2_* resp. *T_2013Q1_* are time dummies equal to 1 from 2012Q2 resp. 2013Q1 onward. EtP denotes Exception-to-Policy mortgages.

|  | **Mortgage share with LTV** | | | | **… with LTI** | | **with** | **Mortgage growth** | | | **credit** |
| --- | --- | --- | --- | --- | --- | --- | --- | --- | --- | --- | --- |
|  | **>90%** | **80-90%** | **66-80%** | **<66%** | **>7.6** | **5.4-7.6** | **EtP** | **total** | **private** | **firms** | **firms** |
|  |  |  |  |  |  |  |  |  |  |  |  |
| **LTV_exp_*T_2012Q2_** | -2.44*** | -0.74 | 2.05 | 1.13 | -0.96 | -1.19 | -3.83 | -0.55 | -0.75 | -0.05 | 0.65 |
|  | (0.36) | (1.12) | (1.33) | (0.87) | (1.27) | (0.81) | (2.42) | (0.42) | (-0.48) | (0.68) | (0.67) |
| **CCyB_exp_* T_2013Q1_** | -0.34*** | -1.55*** | 1.95*** | -0.07 | -0.04 | -1.08 | -0.19 | -0.37* | -0.39 | -0.27 | 0.10 |
|  | (0.08) | (0.25) | (0.53) | (0.42) | (0.32) | (0.69) | (0.70) | (0.20) | (0.24) | (0.22) | (0.22) |
|  |  |  |  |  |  |  |  |  |  |  |  |
| Observations | 600 | 600 | 600 | 600 | 575 | 575 | 600 | 850 | 850 | 850 | 850 |
| R-squared | 0.55 | 0.58 | 0.41 | 0.66 | 0.61 | 0.54 | 0.60 | 0.58 | 0.61 | 0.50 | 0.38 |
| Mean (dep. var.) | 2.55 | 14.52 | 38.30 | 44.63 | 14.88 | 24.59 | 21.25 | 4.61 | 4.61 | 4.81 | 4.09 |
| Std (dep. var.) | 3.09 | 6.63 | 6.80 | 7.73 | 9.42 | 7.26 | 13.58 | 2.41 | 2.75 | 5.08 | 5.92 |
|  |  |  |  |  |  |  |  |  |  |  |  |

Standard errors are determined by a wild cluster bootstrap with ***,**,* denoting significance at the 1%, 5% and 10% levels, respectively.

**Table A.6** Coincidence with increased risk-weight in SA approach: interaction with IRB banks. All regressions control for bank and quarter time fixed effects. *LTV_exp_* and *CCyB*_exp_ measure the standardised treatment intensities of the LTV cap and the CCyB activation. *IRB_dum_* is a dummy for the three banks using the IRB approach.*T_2012Q3_* resp. *T_2013Q1_* are time dummies equal to 1 from 2012Q3 resp. 2013Q1 onward. EtP denotes Exception-to-Policy mortgages.

|  | **Mortgage share with LTV** | | | | **… with LTI** | | **with** | **Mortgage growth** | | | **credit** |
| --- | --- | --- | --- | --- | --- | --- | --- | --- | --- | --- | --- |
|  | **>90%** | **80-90%** | **66-80%** | **<66%** | **>7.6** | **5.4-7.6** | **EtP** | **total** | **private** | **firms** | **firms** |
|  |  |  |  |  |  |  |  |  |  |  |  |
| **LTV_exp_*T_2012Q3_** | -2.25*** | -0.35 | 1.49 | 1.11 | -1.45 | -0.85 | -3.13 | -0.48 | -0.77 | 0.25 | 0.49 |
|  | (0.33) | (1.08) | (1.32) | (0.74) | (1.16) | (0.72) | (2.38) | (0.43) | (0.48) | (0.59) | (0.71) |
| **CCyB_exp_* T_2013Q1_** | -0.35*** | -1.62*** | 2.00*** | -0.03 | 0.07 | -1.00 | -0.53 | -0.38* | -0.36 | -0.40 | 0.09 |
|  | (0.08) | (0.26) | (0.55) | (0.41) | (0.35) | (0.67) | (0.80) | (0.21) | (0.25) | (0.25) | (0.26) |
| **IRBdum* T_2013Q1_** | -0.51 | -1.53 | 1.09 | 0.95 | 2.59 | 1.73 | -7.59 | -0.25 | 0.58 | -2.83** | -0.30 |
|  | (0.84) | (2.59) | (1.95) | (1.99) | (1.88) | (1.59) | (5.25) | (1.14) | (1.25) | (1.18) | (1.39) |
|  |  |  |  |  |  |  |  |  |  |  |  |
| Observations | 600 | 600 | 600 | 600 | 575 | 575 | 600 | 850 | 850 | 850 | 850 |
| R-squared | 0.56 | 0.58 | 0.41 | 0.66 | 0.61 | 0.54 | 0.60 | 0.58 | 0.60 | 0.50 | 0.38 |
| Mean (dep. var.) | 2.55 | 14.52 | 38.30 | 44.63 | 14.88 | 24.59 | 21.25 | 4.61 | 4.61 | 4.81 | 4.09 |
| Std (dep. var.) | 3.09 | 6.63 | 6.80 | 7.73 | 9.42 | 7.26 | 13.58 | 2.41 | 2.75 | 5.08 | 5.92 |
|  |  |  |  |  |  |  |  |  |  |  |  |

Standard errors are determined by a wild cluster bootstrap with ***,**,* denoting significance at the 1%, 5% and 10% levels, respectively.

**Table A.7** Effects of the LTV cap before CCyB activation, sample stops in 2012Q4. All regressions control for bank and quarter time fixed effects. *LTV_exp_* and *CCyB*_exp_ measure the standardised treatment intensities of the LTV cap and the CCyB activation. *T_2012Q3_* is time dummy equal to 1 from 2012Q3 onward. EtP denotes Exception-to-Policy mortgages.

|  | **Mortgage share with LTV** | | | | **…with LTI** | | **with** | **Mortgage growth** | | | **credit** |
| --- | --- | --- | --- | --- | --- | --- | --- | --- | --- | --- | --- |
|  | **>90%** | **80-90%** | **66-80%** | **<66%** | **>7.6** | **5.4-7.6** | **EtP** | **total** | **private** | **firms** | **firms** |
|  |  |  |  |  |  |  |  |  |  |  |  |
| **LTV_exp_*T_2012Q3_** | -1.99*** | -0.35 | 1.04 | 1.30 | 0.41 | -1.64 | -3.06 | -0.35 | -0.46 | 0.28 | 0.29 |
|  | (0.42) | (0.64) | (0.98) | (0.94) | (1.01) | (-1.23) | (-1.98) | (0.38) | (0.33) | (0.89) | (1.22) |
|  |  |  |  |  |  |  |  |  |  |  |  |
| Observations | 175 | 175 | 175 | 175 | 167 | 167 | 175 | 175 | 175 | 175 | 175 |
| R-squared | 0.60 | 0.68 | 0.62 | 0.75 | 0.66 | 0.58 | 0.60 | 0.79 | 0.86 | 0.73 | 0.55 |
| Mean (dep. var.) | 2.55 | 14.52 | 38.30 | 44.63 | 14.88 | 24.59 | 21.25 | 4.61 | 4.61 | 4.81 | 4.09 |
| Std (dep. var.) | 3.09 | 6.63 | 6.80 | 7.73 | 9.42 | 7.26 | 13.58 | 2.41 | 2.75 | 5.08 | 5.92 |
|  |  |  |  |  |  |  |  |  |  |  |  |

Standard errors are determined by a wild cluster bootstrap with ***,**,* denoting significance at the 1%, 5% and 10% levels, respectively.

**Table A.8** Time-varying covariates as additional controls. All regressions control for bank and quarter time fixed effects. The following time-varying covariates are included: cost-income ratio, return-on-assets, trading assets/assets, deposits/assets, loan losses, funding cost, commission income/operating income, trading income/ operating income. Most of them are not statistically significant at conventional levels. Exceptions are funding costs and trading income over operating income. *LTV_exp_* and *CCyB*_exp_ measure the standardised treatment intensities of the LTV cap and the CCyB activation. *T_2012Q3_* resp. *T_2013Q1_* are time dummies equal to 1 from 2012Q3 resp. 2013Q1 onward. EtP denotes Exception-to-Policy mortgages.

|  | **Mortgage share with LTV** | | | | **… with LTI** | | **with** | **Mortgage growth** | | | **credit** |
| --- | --- | --- | --- | --- | --- | --- | --- | --- | --- | --- | --- |
|  | **>90%** | **80-90%** | **66-80%** | **<66%** | **>7.6** | **5.4-7.6** | **EtP** | **total** | **private** | **firms** | **firms** |
|  |  |  |  |  |  |  |  |  |  |  |  |
| **LTV_exp_*T_2012Q3_** | -2.28*** | -0.47 | 1.51 | 1.23* | -0.87 | -0.84 | -3.66* | -0.53 | -0.73* | -0.02 | 0.67 |
|  | (0.32) | (1.16) | (1.39) | (0.68) | (1.19) | (0.71) | (2.11) | (0.38) | (0.39) | (0.66) | (0.68) |
| **CCyB_exp_* T_2013Q1_** | -0.31*** | -1.68*** | 1.81*** | 0.19 | -0.02 | -0.93 | 0.07 | -0.45* | -0.41 | -0.42 | -0.10 |
|  | (0.11) | (0.25) | (0.45) | (0.34) | (0.40) | (0.85) | (0.63) | (0.23) | (0.28) | (0.25) | (0.25) |
|  |  |  |  |  |  |  |  |  |  |  |  |
| Observations | 576 | 576 | 576 | 576 | 551 | 551 | 576 | 816 | 816 | 816 | 816 |
| R-squared | 0.57 | 0.57 | 0.42 | 0.66 | 0.65 | 0.57 | 0.61 | 0.61 | 0.64 | 0.51 | 0.39 |
| Mean (dep. var.) | 2.55 | 14.52 | 38.30 | 44.63 | 14.88 | 24.59 | 21.25 | 4.61 | 4.61 | 4.81 | 4.09 |
| Std (dep. var.) | 3.09 | 6.63 | 6.80 | 7.73 | 9.42 | 7.26 | 13.58 | 2.41 | 2.75 | 5.08 | 5.92 |
|  |  |  |  |  |  |  |  |  |  |  |  |

Standard errors are determined by a wild cluster bootstrap with ***,**,* denoting significance at the 1%, 5% and 10% levels, respectively.

**Table A.9** Cantonal market shares interacted with time dummies as additional controls. All regressions control for bank and quarter time fixed effects. Each bank’s cantonal market share in eight small cantons (with either a high LTV or CCyB treatment intensity) interacted with the ex post time dummies are included as additional controls. *LTV_exp_* and *CCyB*_exp_ measure the standardised treatment intensities of the LTV cap and the CCyB activation. *T_2012Q3_* resp. *T_2013Q1_* are time dummies equal to 1 from 2012Q3 resp. 2013Q1 onward. EtP denotes Exception-to-Policy mortgages.

|  | **Mortgage share with LTV** | | | | **…with LTI** | | **with** | **Mortgage growth** | | | **credit** |
| --- | --- | --- | --- | --- | --- | --- | --- | --- | --- | --- | --- |
|  | **>90%** | **80-90%** | **66-80%** | **<66%** | **>7.6** | **5.4-7.6** | **EtP** | **total** | **private** | **firms** | **firms** |
|  |  |  |  |  |  |  |  |  |  |  |  |
| **LTV_exp_*T_2012Q3_** | -2.14*** | -1.18 | 1.97 | 1.35 | -0.57 | 0.16 | -2.24 | -0.61 | -0.62 | -1.01 | -0.22 |
|  | (0.40) | (1.30) | (1.51) | (1.08) | (1.83) | (0.96) | (3.32) | (0.51) | (0.45) | (0.73) | (1.07) |
| **CCyB_exp_* T_2013Q1_** | -0.34*** | -2.00*** | 2.50*** | -0.16 | 0.26 | -0.91 | -0.40 | -0.78** | -0.84 | -0.54* | 0.08 |
|  | (0.11) | (0.37) | (0.75) | (0.53) | (0.48) | (0.69) | (1.09) | (0.42) | (0.61) | (0.32) | (0.34) |
|  |  |  |  |  |  |  |  |  |  |  |  |
| Observations | 600 | 600 | 600 | 600 | 575 | 575 | 600 | 850 | 850 | 850 | 850 |
| R-squared | 0.57 | 0.59 | 0.43 | 0.67 | 0.62 | 0.55 | 0.61 | 0.62 | 0.64 | 0.51 | 0.39 |
| Mean (dep. var.) | 2.55 | 14.52 | 38.30 | 44.63 | 14.88 | 24.59 | 21.25 | 4.61 | 4.61 | 4.81 | 4.09 |
| Std (dep. var.) | 3.09 | 6.63 | 6.80 | 7.73 | 9.42 | 7.26 | 13.58 | 2.41 | 2.75 | 5.08 | 5.92 |
|  |  |  |  |  |  |  |  |  |  |  |  |

Standard errors are determined by a wild cluster bootstrap with ***,**,* denoting significance at the 1%, 5% and 10% levels, respectively.

**Table A.10** Comparison to Auer and Ongena (2019). All regressions control for bank and quarter time fixed effects. *LTV_exp_* and *CCyB*_exp_ measure the standardised treatment intensities of the LTV cap and the CCyB activation. *T_2012Q3_* resp. *T_2013Q1_* are time dummies equal to 1 from 2012Q3 resp. 2013Q1 onward. EtP denotes Exception-to-Policy mortgages.

|  | **Mortgage share with LTV** | | | | **… with LTI** | | **with** | **Mortgage growth** | | | **credit** |
| --- | --- | --- | --- | --- | --- | --- | --- | --- | --- | --- | --- |
|  | **>90%** | **80-90%** | **66-80%** | **<66%** | **>7.6** | **5.4-7.6** | **EtP** | **total** | **private** | **firms** | **firms** |
|  |  |  |  |  |  |  |  |  |  |  |  |
| **20 banks (bank sample as in Auer and Ongena)** | | | | | | | | | | | |
| **LTV_exp_*T_2012Q3_** | -2.44*** | -1.28 | 1.81 | 1.91* | -1.94 | -1.02 | -3.10 | -0.58 | -0.64 | -0.74 | 0.32 |
|  | (0.30) | (1.38) | (1.64) | (1.07) | (1.60) | (0.96) | (2.97) | (0.45) | (0.48) | (0.62) | (0.85) |
| **CCyB_exp_*T_2013Q1_** | -0.32*** | -1.48*** | 1.82*** | -0.02 | 0.09 | -0.93 | -0.30 | -0.26 | -0.28 | -0.24 | 0.04 |
|  | (0.06) | (0.27) | (0.44) | (0.30) | (0.33) | (0.64) | (0.66) | (0.15) | (0.19) | (0.22) | (0.24) |
|  |  |  |  |  |  |  |  |  |  |  |  |
| Observations | 480 | 480 | 480 | 480 | 456 | 456 | 480 | 680 | 680 | 680 | 680 |
| R-squared | 0.59 | 0.57 | 0.43 | 0.65 | 0.63 | 0.51 | 0.65 | 0.62 | 0.63 | 0.45 | 0.32 |
| Mean (dep. var.) | 2.223 | 14.54 | 38.70 | 44.54 | 14.80 | 25.21 | 20.38 | 4.440 | 4.632 | 4.112 | 3.392 |
| Std (dep. var.) | 2.725 | 6.557 | 6.739 | 7.475 | 9.122 | 6.760 | 13.34 | 2.359 | 2.724 | 4.607 | 5.709 |
|  |  |  |  |  |  |  |  |  |  |  |  |
| **2012Q3-2013Q4 (sample period as in Auer and Ongena)** | | | | | | | | | | | |
| **CCyB_exp_*T_2013Q1_** | -0.29*** | -1.70*** | 1.87*** | 0.12 | -0.39* | -1.06** | 1.02 | -0.08 | 0.01 | -0.39** | 0.16 |
|  | (0.10) | (0.21) | (0.23) | (0.20) | (0.23) | (0.59) | (0.69) | (0.10) | (0.10) | (0.18) | (0.36) |
|  |  |  |  |  |  |  |  |  |  |  |  |
| Observations | 150 | 150 | 150 | 150 | 144 | 144 | 150 | 150 | 150 | 150 | 150 |
| R-squared | 0.55 | 0.76 | 0.63 | 0.81 | 0.67 | 0.60 | 0.68 | 0.85 | 0.84 | 0.88 | 0.75 |
| Mean (dep. var.) | 2.613 | 14.95 | 37.08 | 45.36 | 13.09 | 24.04 | 19.90 | 4.581 | 4.421 | 5.291 | 4.159 |
| Std (dep. var.) | 2.794 | 6.622 | 6.093 | 8.070 | 8.678 | 6.908 | 13.57 | 2.136 | 2.140 | 5.320 | 5.799 |
|  |  |  |  |  |  |  |  |  |  |  |  |
| **treatment definition: above median residential risk weighted assets/domestic assets (as in Auer and Ongena)** | | | | | | | | | | | |
| **LTV_exp_*T_2012Q3_** | -2.15*** | -0.58 | 1.53 | 1.20* | -1.21 | -0.85 | -4.67** | -0.50 | -0.75 | 0.17 | 0.49 |
|  | (0.28) | (1.07) | (1.26) | (0.64) | (1.09) | (0.72) | (2.13) | (0.45) | (0.47) | (0.57) | (0.60) |
| **RRWA/assets*T_2013Q1_** | 1.04* | -0.41 | -0.64 | 0.01 | 0.05 | -1.09 | -5.82 | 0.12 | -0.14 | 1.26 | 0.17 |
|  | (0.53) | (1.66) | (1.92) | (1.33) | (2.22) | (1.37) | (3.40) | (0.79) | (1.00) | (1.22) | (1.35) |
|  |  |  |  |  |  |  |  |  |  |  |  |
| Observations | 600 | 600 | 600 | 600 | 575 | 575 | 600 | 850 | 850 | 850 | 850 |
| R-squared | 0.56 | 0.57 | 0.39 | 0.66 | 0.61 | 0.54 | 0.60 | 0.57 | 0.60 | 0.50 | 0.38 |
| Mean (dep. var.) | 2.55 | 14.52 | 38.30 | 44.63 | 14.88 | 24.59 | 21.25 | 4.61 | 4.61 | 4.81 | 4.09 |
| Std (dep. var.) | 3.09 | 6.63 | 6.80 | 7.73 | 9.42 | 7.26 | 13.58 | 2.41 | 2.75 | 5.08 | 5.92 |
|  |  |  |  |  |  |  |  |  |  |  |  |

Standard errors are determined by a wild cluster bootstrap with ***,**,* denoting significance at the 1%, 5% and 10% levels, respectively.

**Table A.11** Comparison to Basten (2020). All regressions control for bank and quarter time fixed effects. *LTV_exp_* and *CCyB*_exp_ measure the standardised treatment intensities of the LTV cap and the CCyB activation. *T_2012Q3_* resp. *T_2013Q1_* are time dummies equal to 1 from 2012Q3 resp. 2013Q1 onward. EtP denotes Exception-to-Policy mortgages.

|  | **Mortgage share with LTV** | | | | **…with LTI** | | **with** | **Mortgage growth** | | | **credit** |
| --- | --- | --- | --- | --- | --- | --- | --- | --- | --- | --- | --- |
|  | **>90%** | **80-90%** | **66-80%** | **<66%** | **>7.6** | **5.4-7.6** | **EtP** | **total** | **private** | **firms** | **firms** |
|  |  |  |  |  |  |  |  |  |  |  |  |
| **7 banks (bank sample as in Basten)** | | | | | | | | | | | |
| **LTV_exp_*T_2012Q3_** | -2.03*** | -0.23 | 0.33 | 1.93** | -1.75 | -1.13** | -5.43* | -0.02 | -0.43 | 0.49 | 0.88* |
|  | (0.19) | (0.50) | (0.61) | (0.63) | (1.75) | (0.41) | (2.64) | (0.39) | (0.60) | (0.75) | (0.40) |
| **CCyB_exp_*T_2013Q1_** | -3.19** | -7.23* | 23.02*** | -12.60*** | -5.47 | -0.96 | 2.06 | -7.10*** | -6.61* | -8.05*** | -1.31 |
|  | (1.14) | (3.72) | (6.33) | (3.41) | (7.89) | (4.30) | (15.69) | (1.59) | (2.87) | (2.14) | (3.50) |
|  |  |  |  |  |  |  |  |  |  |  |  |
| Observations | 216 | 216 | 216 | 216 | 216 | 216 | 216 | 306 | 306 | 306 | 306 |
| R-squared | 0.63 | 0.59 | 0.55 | 0.71 | 0.54 | 0.62 | 0.59 | 0.66 | 0.71 | 0.50 | 0.26 |
| Mean (dep. var.) | 2.653 | 12.83 | 38.81 | 45.70 | 12.48 | 22.20 | 18.16 | 4.811 | 5.346 | 3.485 | 2.830 |
| Std (dep. var.) | 3.318 | 5.963 | 7.349 | 7.131 | 8.133 | 7.238 | 12.38 | 2.019 | 3.029 | 4.529 | 5.209 |
|  |  |  |  |  |  |  |  |  |  |  |  |
| **2012Q3-2013Q3 (sample period as in Basten)** | | | | | | | | | | | |
| **CCyB_exp_*T_2013Q1_** | -0.33*** | -1.66*** | 1.85*** | 0.15 | -0.37 | -1.01** | 1.14 | -0.10 | 0.01 | -0.42* | 0.15 |
|  | (0.09) | (0.21) | (0.23) | (0.18) | (0.24) | (0.57) | (0.67) | (0.11) | (0.08) | (0.22) | (0.33) |
|  |  |  |  |  |  |  |  |  |  |  |  |
| Observations | 125 | 125 | 125 | 125 | 120 | 120 | 125 | 125 | 125 | 125 | 125 |
| R-squared | 0.57 | 0.76 | 0.66 | 0.83 | 0.66 | 0.62 | 0.68 | 0.87 | 0.88 | 0.92 | 0.79 |
| Mean (dep. var.) | 2.800 | 14.98 | 37.27 | 44.95 | 12.90 | 24.21 | 20.05 | 4.612 | 4.456 | 5.246 | 4.132 |
| Std (dep. var.) | 2.845 | 6.646 | 6.082 | 7.667 | 8.349 | 7.046 | 13.88 | 2.119 | 2.092 | 5.317 | 5.773 |
|  |  |  |  |  |  |  |  |  |  |  |  |
| **treatment definition: below median capital cushion and above median mortgage/assets (as in Basten)** | | | | | | | | | | | |
| **LTV_exp_*T_2012Q3_** | -2.32*** | -0.58 | 1.76 | 1.14 | -1.23 | -0.74 | -3.91 | -0.55 | -0.76* | -0.04 | 0.47 |
|  | (0.30) | (1.15) | (1.23) | (0.69) | (1.17) | (0.75) | (2.39) | (0.39) | (0.42) | (0.62) | (0.61) |
| **Basten*T_2012Q3_** | -0.93* | -3.81* | 7.85*** | -3.11 | -0.39 | -1.68 | -5.87* | -2.01*** | -2.06*** | -1.36* | 0.07 |
|  | (0.51) | (2.01) | (1.12) | (2.00) | (1.32) | (2.50) | (3.37) | (0.52) | (0.64) | (0.78) | (0.72) |
|  |  |  |  |  |  |  |  |  |  |  |  |
| Observations | 600 | 600 | 600 | 600 | 575 | 575 | 600 | 850 | 850 | 850 | 850 |
| R-squared | 0.56 | 0.57 | 0.42 | 0.66 | 0.61 | 0.54 | 0.60 | 0.59 | 0.61 | 0.50 | 0.38 |
| Mean (dep. var.) | 2.55 | 14.52 | 38.30 | 44.63 | 14.88 | 24.59 | 21.25 | 4.61 | 4.61 | 4.81 | 4.09 |
| Std (dep. var.) | 3.09 | 6.63 | 6.80 | 7.73 | 9.42 | 7.26 | 13.58 | 2.41 | 2.75 | 5.08 | 5.92 |
|  |  |  |  |  |  |  |  |  |  |  |  |

Standard errors are determined by a wild cluster bootstrap with ***,**,* denoting significance at the 1%, 5% and 10% levels, respectively.
